# Quantitative Method for Analysis of Lipids by LC-HRMS and Fatty Acid Methyl Ester by GC-FID in Macauba (*Acrocomia aculeata*) Oils

**DOI:** 10.3390/plants15020268

**Published:** 2026-01-15

**Authors:** Eva Zopelario S. Ferro, Ana Laura M. Brand, Ricardo Sposina S. Teixeira, Claudia M. Rezende

**Affiliations:** 1 Aroma Analysis Laboratory, Institute of Chemistry, Federal University of Rio de Janeiro, Rio de Janeiro 21941-909, RJ, Brazil; 2 Bioethanol Laboratory, Institute of Chemistry, Federal University of Rio de Janeiro, Rio de Janeiro 21941-596, RJ, Brazil; 3 Metabolomics Laboratory (LabMeta-LADETEC), Institute of Chemistry, Federal University of Rio de Janeiro, Rio de Janeiro 21941-598, RJ, Brazil

**Keywords:** *Acrocomia aculeata*, cerrado fruit, quality control, lipids, chemometrics

## Abstract

Macauba (*Acrocomia aculeata*) is a promising source of vegetable oils with distinct applications for its pulp and seed fractions. This study presents the first comprehensive quantitative analysis of eleven commercial macauba oils available in the Brazilian market, using validated methods of GC-FID and LC-HRMS. The analysis revealed significant variability among samples. Control pulp oils (PCCs) were characterized by the predominance of oleic acid (C18:1) and palmitic acid (C16:0) methyl esters, and TG 54:3 was the major lipid species, reaching up to 12.11 g 100 g oil^−1^. For control seed oils (SCCs), the profile was dominated by lauric acid (C12:0) and oleic acid methyl ester; TG 36:0 was the most abundant lipid, which reached concentrations of 49.20 g 100 g oil^−1^. Among commercial samples, PC3 followed the expected pulp oil profile, whereas PC4 showed deviations. Others commercial samples (PC2, PC5, SC3, SC4, SC5) deviated significantly from expected profiles, showing high levels of linoleic acid (C18:2), and predominance of TG 54:6, with concentrations reaching 61.74 g 100 g oil^−1^. The integrated GC-FID and LC-HRMS approach provides robust, sensitive, and discriminative analysis of FAMEs and lipid composition of macauba oil samples. These methodologies are essential for quality control in the food and bioproduct sectors, ensuring the chemical integrity of macauba commercial oils.

## 1. Introduction

The macauba palm (*Acrocomia aculeata* (Jacq.) Lodd. ex Mart., Arecaceae) is native to tropical and subtropical regions of Central and South America, with a wide geographical distribution [1]. Brazil represents the main area of occurrence, with the species being found across nearly the entire national territory, except for the southern region [2]. Macauba can be most found in the Cerrado biome, especially in the states of Minas Gerais, Goiás, Mato Grosso, and Mato Grosso do Sul [2,3]. Due to its broad distribution, the species is known in Brazil by several different names, including macauba, bocaiuva, coco-catarro, mucaja, macaiba, and umbocaiuva [2]. The genus *Acrocomia* presents taxonomic variation, with classifications recognizing between two and eight species, of which *A. aculeata*, *A. totai*, and *A. intumescens* hold the greatest economic interest [1,2,4].

The species *A. aculeata* (macauba) has gained increasing attention in global markets, primarily due to its potential as a source of vegetable oil and as a raw material for the bioenergy sector, thereby representing both socioeconomic and scientific interest [1,5,6]. This growing interest is largely attributed to its high productivity, estimated at 25 tons of fruit per hectare per year. This corresponds to over 6 tons of oil per hectare per year, a yield comparable only to that of palm oil (*Elaeis guineensis*) [5].

The macauba fruit consists of epicarp (shell), mesocarp (pulp), endocarp, and kernel (seed). Both the pulp (mesocarp) and the seed have high oil content, with distinct lipid profiles [7]. While pulp oil is rich in oleic acid (C18:1) and palmitic acid (C16:0), the seed oil is rich in lauric acid (C12:0) and oleic acid [7]. Due to these two distinct lipid compositions, the macauba fruit is an attractive asset for a wide range of industrial applications across multiple sectors [8,9,10,11]. For the species *A. totai* and *A. intumescens,* the pulp and seed oil exhibit a similar fatty acid and chemical profile to *A. aculeata* [12,13,14].

Macauba is highly adaptable to different ecosystems, including nutrient-poor sandy soils with limited water availability and marked dry seasons, characteristic of the Brazilian Cerrado [3,15,16]. It can also be cultivated in agroforestry systems intercropped with pasturelands and other crops such as coffee, corn, and beans, which can be incorporated in an agrosilvopastoral system. It is also employed in programs for the restoration of degraded areas [3,17].

Beyond its potential for energy applications, the macauba fruit stands out for its chemical composition, which makes it a versatile raw material for the food, cosmetic, and pharmaceutical industries [18,19,20]. In regions where the species occurs naturally, macauba oils are traditionally used for human consumption, and the fruit itself is consumed fresh or processed into products such as ice cream, liqueurs, and juices [21].

Pulp oil is often compared to olive oil due to their similar fatty acid methyl esters (FAMEs) profiles, rich in oleic (C18:1) and palmitic (C16:0) acids [10,22]. In addition to its fatty acid composition, pulp oil contains significant lipids, such as carotenoids and tocopherols, which exhibit antioxidant activity and contribute to human health benefits. These bioactive compounds further highlight the potential of macauba oils for pharmaceutical applications [8,10,23,24]. Seed oil, on the other hand, is widely compared to coconut oil because of its high content of lauric (C12:0) and myristic (C14:0) acids [20,25]. Due to the abundance of short- and medium-chain fatty acids, in addition to oleic acid (C18:0), seed oil presents greater viscosity than pulp oil, which is of value in the cosmetics industry [3,8].

The quality of a vegetable oil is intrinsically linked to its chemical composition, especially the proportions of free fatty acids (FFAs), phospholipids (PLs), mono- (MGs), di- (DGs), and, most importantly, triacylglycerols (TGs). TGs represent the predominant lipid fraction of the macauba fruit, accounting for approximately 78% of pulp oil and 98% of seed oil [26,27]. Furthermore, they are responsible for the physicochemical properties of these oils, their stability, and industrial applications.

Despite their importance, most research articles report only general FAME profiles or simplified lipid compositions, only in terms of TG profile, for macauba pulp and seed oils, without providing quantitative data. Furthermore, determining the profile of commercial macauba oils is important for identifying variations or detecting possible contaminations, especially given the current lack of studies on the oils available in the Brazilian market.

For the complete characterization of macauba oils, it is appropriate to use a combination of gas chromatography coupled with mass spectrometry (GC-MS) and coupled with flame ionization detector (GC-FID), as well as liquid chromatography coupled with high-resolution mass spectrometry (LC-HRMS), as each technique provides complementary information about lipid composition. The analysis of fatty acids by GC-FID requires their conversion into fatty acid methyl esters (FAMEs), which is a well-established and widely used technique due to its high separation efficiency, repeatability, and sensitivity, making it a reliable method for quantitative analysis [28]. Most studies on the lipid profile of macauba oils use this strategy and report as FAMEs analyzed by GC-FID only as qualitative data. Therefore, this approach remains essential for comparative purposes within the literature.

LC-HRMS is a technique that does not require elaborate sample preparation and has fewer analytical limitations than GC, since the sample does not need to be volatile, making it a more comprehensive approach. LC-HRMS enables the identification of individual lipid species and the determination of exact masses, offering a robust approach for detailed lipid composition studies [29,30]. Therefore, FAME analysis by GC-FID not only facilitates comparison with the existing literature but also serves to corroborate the results obtained from the more comprehensive lipid profiling performed by LC-HRMS.

For these reasons, the primary objective of this study was to develop and validate a robust and quantitative analytical approach based on LC-HRMS and GC-MS/GC-FID techniques for the comprehensive characterization of lipid classes in macauba oils. We hypothesized that this integrated methodology would allow the reliable differentiation of macauba oils according to their compositional features and provide a solid basis for quality assessment.

As a secondary objective, the validated methods were applied to macauba oil samples obtained from different suppliers in the Brazilian market to evaluate compositional variability under real commercial conditions. Fatty acid methyl ester profiles were determined by GC-MS and quantified by GC-FID, while individual lipid species were annotated and quantified by LC-HRMS. These results were compared with the literature data for macauba and other commercial oils to contextualize the observed variations. This application allowed the discussion of quality-related differences and the identification of compositional patterns that may indicate deviations from expected profiles.

## 2. Results

### 2.1. Methods Validation

The fatty acid methyl ester (FAME) quantification and LC-HRMS methods were validated according to ANVISA (Agência Nacional de Vigilância Sanitária-Brazil) and FDA (Food and Drug Administration–United States of America) standards [31,32]. For the validation, a mix of all samples was used. All the parameters obtained for both methods can be found in Table 1. For the FAME method by GC-FID, the analytical curve for methyl *n*-heptadecanoate showed a high R^2^ value, above 0.99. Methyl *n*-heptadecanoate was chosen as an internal analytical standard as heptanoic acid was not found in the analyzed samples.

**Figure 1:**
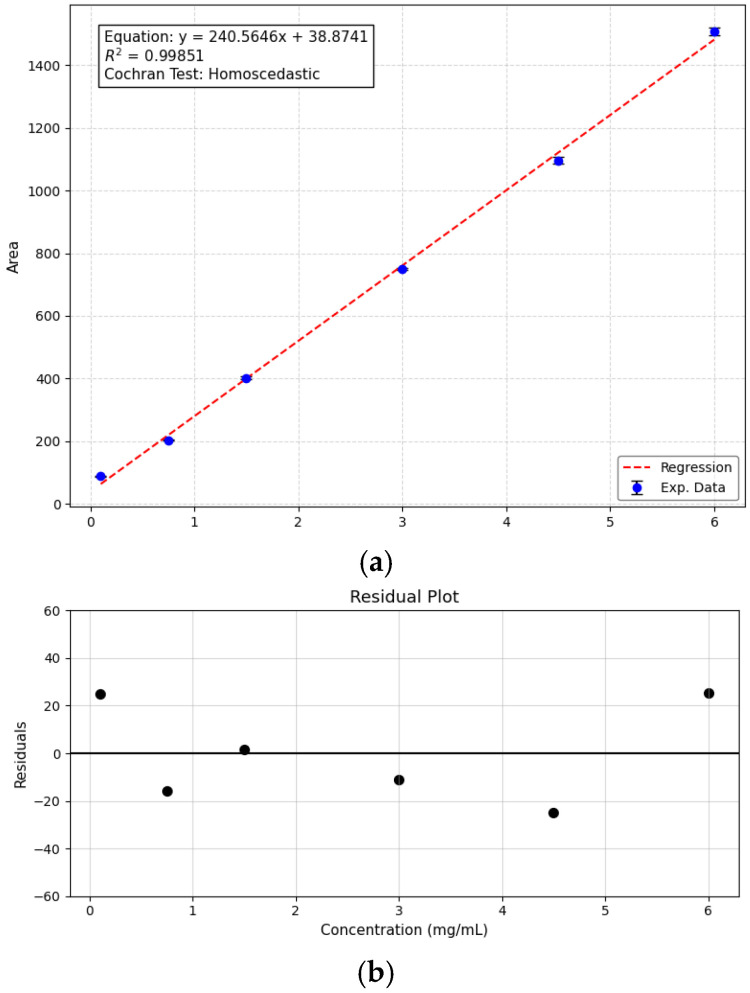
(**a**) Analytical curve for FAME quantification in macauba (*Acrocomia aculeata*) oils by GC-FID using methyl *n*-heptadecanoate in six different concentrations. (**b**) Residual plot for analytical curve for FAME quantification in macauba oils. The random dispersion of residuals around the zero line indicates homoscedasticity, meaning the error variance is constant.

The calibration curve for the FAME method, using methyl *n*-heptadecanoate as a standard, can be found in Figure 1. Furthermore, carryover and matrix effect were not observed in the developed method.

**Figure 2:**
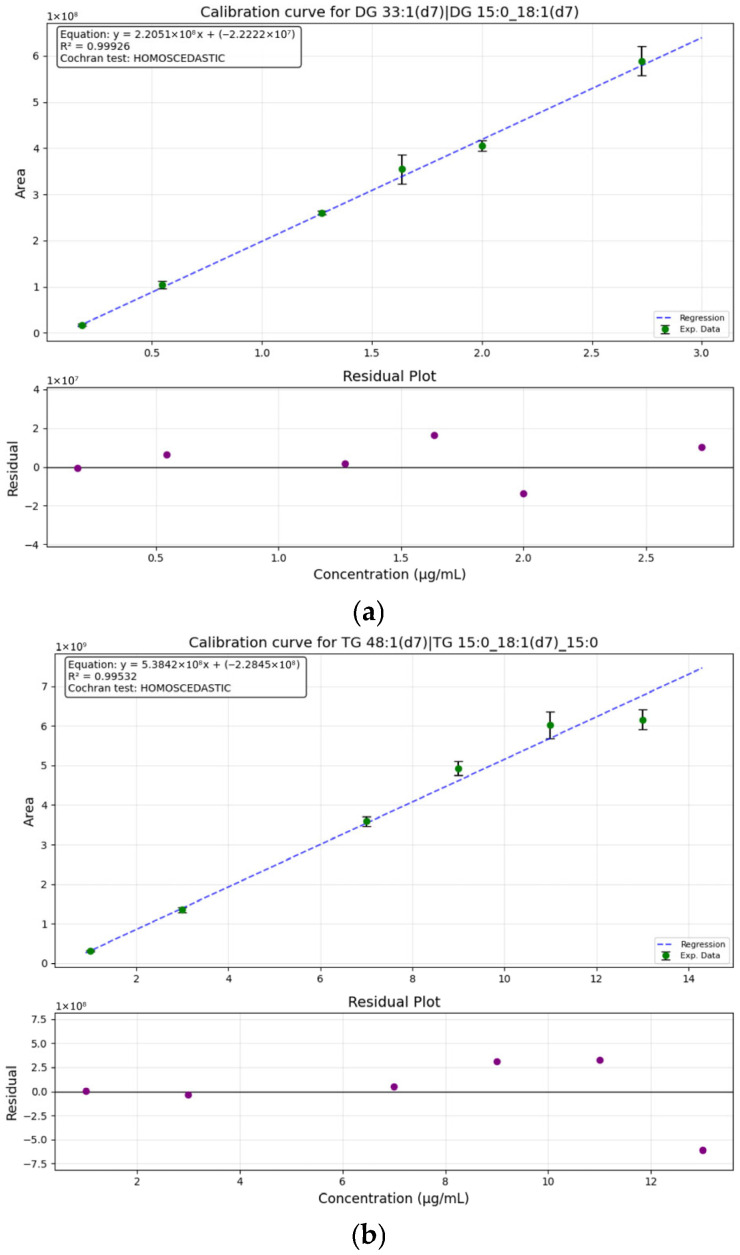
Calibration curve for lipid quantification in macauba (*Acrocomia aculeata*) oils by LC-HRMS using DG 33:1(d7)|DG 15:0_18:1(d7) (**a**) and TG 48:1(d7)|TG 15:0_18:1(d7)_15:0 (**b**) in six different concentrations. Both with the residual plot for the respective curve. The random dispersion of residuals around the zero line indicates homoscedasticity, meaning the error variance is constant.

As for the LC-HRMS analysis, the validation was performed using a commercial mix of deuterated lipids (SPLASH™ Lipidomix™). Even though this mix provides analytical standards for many different lipid classes, such as phospholipids and cholesterol esters, the validation was only performed for lipid classes found in the macauba oils (TG and DG). Thus, the calibration curves for DG 33:1(d7)|DG 15:0_18:1(d7) and TG 48:1(d7)|TG 15:0_18:1(d7)_15:0 can be found in Figure 2. Both showed a high R^2^ value, above 0.99. Additionally, carryover and matrix effect were not observed in the developed method for both lipid classes.

### 2.2. FAME in Macauba Oils

The FAME composition of macauba pulp and seed oils, determined by GC-FID, is presented in Table 2. The seed and pulp oils used as control samples, from the northern part of Minas Gerais and from the same harvest (PCC1_1—pulp oil, and SCC1_1—seed oil), were analyzed in triplicate. The oils presented clearly distinct FAME profiles. The seed oil is mainly composed of methyl ester of lauric acid (33.55%) and oleic acid (36.02%), whereas the pulp oil predominantly contains methyl ester of palmitic acid (20.07%) and oleic acid (59.19%), in agreement with what is reported in the scientific literature [3,4,10,33]. Furthermore, macauba seed oil presents a higher proportion of short-chain fatty acid methyl esters, such as caprylic (C8:0), capric (C10:0), lauric (C12:0), and myristic (C14:0) acids. These fatty acids are absent in the pulp oil, which instead contains methyl ester of palmitoleic (C16:1) and linolenic (C18:3) acids, which are not found in the seed oil.

#### 2.2.1. FAME in Commercial Pulp Oils

**Figure 3:**
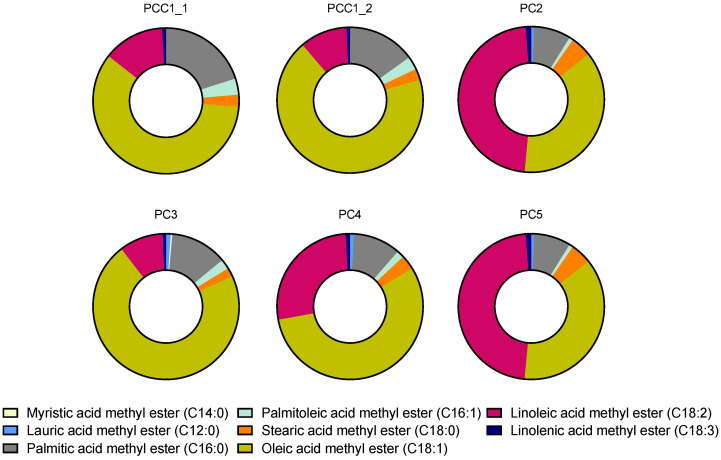
Profile of fatty acid methyl esters from commercial macauba *(Acrocomia aculeata)* pulp oil as percentage content.

The six commercial macauba pulp oil samples showed variations in the relative content of fatty acids among samples (Figure 3). Although all samples contained the same FAMEs, their proportions differed. The FAME profiles of the control samples PCC1_1 and PCC1_2 are in accordance with what is described in the literature [4,9,10,23]. Pulp samples PCC1_1, PCC1_2, PC3, and PC4 exhibited similar compositions, with methyl ester of oleic acid (C18:1) contents of 59.25%, 68.27%, 79.71%, and 56.48%, respectively. The palmitic acid (C16:0) contents for these samples were 20.06%, 15.03%, 14.09%, and 10.60%, respectively. Furthermore, methyl ester of linoleic acid (C18:2) was among the main components that showed significant variation among samples, corresponding to 13.58%, 10.48%, 10.85%, and 27.17%, respectively. The PC4 pulp sample presented the highest linoleic acid (C18:2) content.

Pulp samples PC2 and PC5 exhibited a more distinct FAME profile compared with the other oil samples, with higher methyl ester of linoleic acid (C18:2) content of 47.88% and 47.86%, respectively. In contrast, these samples showed lower methyl ester of oleic acid (C18:1) content (37.58% for PC2 and 37.62% for PC5) and palmitic acid (C16:0) content (8.26% for PC2 and 8.23% for PC5). Additionally, pulp samples PC2, PC3, PC4, and PC5 presented small amounts of short-chain fatty acid methyl esters, such as lauric acid (C12:0), ranging from 0.60% to 1.20%. Additionally, myristic acid (C14:0) was detected only in the sample PC3 (0.37%).

The results of the quantification of FAMEs in these six commercial macauba pulp oils are presented in Table 3. Short-chain FAMEs, which appeared only in samples PC2, PC3, PC4, and PC5, were detected below the quantification limit of the method, thus could not be quantified. A similar situation was observed for linolenic acid methyl ester (C18:3) in all samples and for palmitoleic acid methyl esters (C16:1) in PC2, PC4, and PC5.

The most abundant FAMEs in all pulp oil samples were palmitic acid methyl ester (C16:0) and oleic acid methyl ester (C18:1). Samples PCC1_1 and PCC1_2 exhibited the highest C16:0 levels, with 12.99 and 11.59 g g oil^−1^, respectively, whereas samples PC2 and PC5 showed the lowest concentrations, with 6.74 and 6.28 g g oil^−1^, respectively. The highest concentrations of C18:1 were observed in samples PCC1_2, PC3, and PC4, with 52.76, 56.30, and 49.15 g g oil^−1^, respectively.

Furthermore, the samples PC2, PC4, and PC5 displayed high amounts of linoleic acid methyl ester (C18:2) (39.10, 23.64, and 36.51 g g oil^−1^, respectively). However, in samples PCC1_1, PCC1_2, and PC3, this FAME was present in low concentrations, in the range of 7.66–8.80 g g oil^−1^. Stearic acid methyl ester (C18:0) showed low concentrations across all samples, ranging from 1.73 to 3.84 g g oil^−1^, with PC2 and PC5 exhibiting the highest values and PCC1_1 and PCC1_2 the lowest. For PC3, the concentration of C18:0 was below the quantification limit.

#### 2.2.2. FAME in Commercial Seed Oil

**Figure 4:**
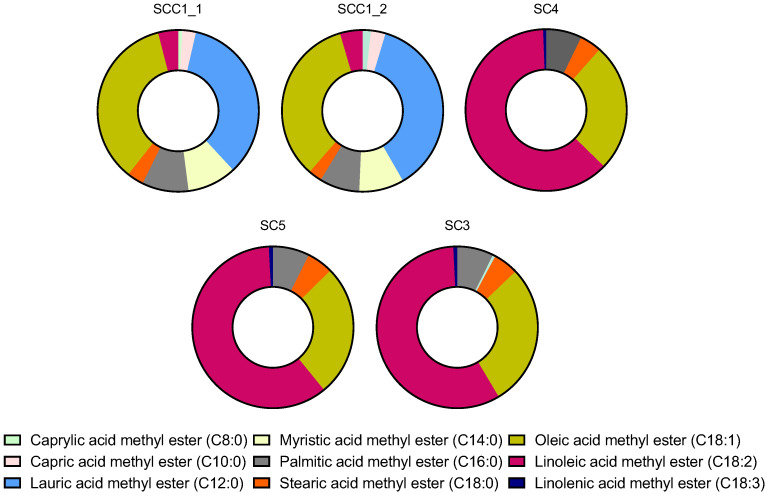
Profile of fatty acid methyl esters from commercial macauba *(Acrocomia aculeata)* seed oil as percentage content.

The FAME composition of five commercial macauba seed oils is shown in Figure 4. The samples exhibited differences in their fatty acid methyl ester profiles. All samples contained methyl ester of palmitic acid (C16:0), stearic acid (C18:0), oleic acid (C18:1), and linoleic acid (C18:2). However, only seed samples SCC1_1 and SCC1_2 contained short-chain fatty acids such as methyl ester of caprylic acid (C8:0) at 0.82% and 1.67%, capric acid (C10:0) at 2.75% and 2.98%, and myristic acid (C14:0) at 9.81% and 8.07%., Lauric acid methyl ester (C12:0) was the most abundant low-chain FAME in both samples (34.35% and 37.14%, respectively), in accordance with the literature [8,10,26]. In contrast, only seed oil samples SC4, SC5, and SC3 exhibited methyl ester of linolenic acid (C18:3), with contents of 0.58%, 0.77%, and 0.76%, respectively.

The FAME quantification determined by GC-FID for the commercial macauba seed oil samples is presented in Table 4. The following FAMES were found under the quantification limit and therefore were not quantified: Caprylic acid methyl ester (C8:0), detected only in SCC1_1; palmitoleic acid methyl ester (C16:1), detected only in SC3; and linolenic acid methyl ester (C18:3), detected only in SC4, SC5, and SC3.

Short-chain fatty acid methyl esters were observed only in samples SCC1_1 and SCC1_2, and showed statistically similar values. For capric acid methyl ester (C10:0), SCC1_1 and SCC1_2 contained 2.63 and 2.82 g g oil^−1^, respectively. Myristic acid methyl ester (C14:0) appeared at higher concentrations, with 9.53 and 8.51 g g oil^−1^, respectively. Lauric acid methyl ester (C12:0) was among the most abundant components in these samples, with concentrations of 33.03 and 35.02 g g oil^−1^ for SCC1_1 and SCC1_2, respectively.

Palmitic acid methyl ester (C16:0) was detected in all samples, showing relatively close values across them. SCC1_1 presented the highest concentration (8.88 g g oil^−1^), whereas SC3 showed the lowest (6.19 g g oil^−1^). Stearic acid methyl ester (C18:0) was also present in all samples in the range of 2.45–4.64 g g oil^−1^.

Oleic acid methyl ester (C18:1), together with lauric acid mehyl ester (C12:0), were showed the highest concentrations in samples SCC1_1 and SCC1_2, with 33.56 and 31.77 g g oil^−1^, respectively. Samples SC4, SC5, and SC3 also contained substantial amounts of C18:1 (29.41, 23.11, and 24.05 g g oil^−1^, respectively). Linoleic acid methyl ester (C18:2) was the predominant component in samples SC4, SC5, and SC3, with concentrations of 69.65, 52.89, and 48.72 g g oil^−1^, respectively. In contrast, SCC1_1 and SCC1_2 exhibited the lowest C18:2 concentrations, with 3.80 and 4.18 g g oil^−1^.

### 2.3. Comprehensive Lipid Analysis in Commercial Macauba Oils by LC-HRMS

#### 2.3.1. Lipid Profile

**Figure 5:**
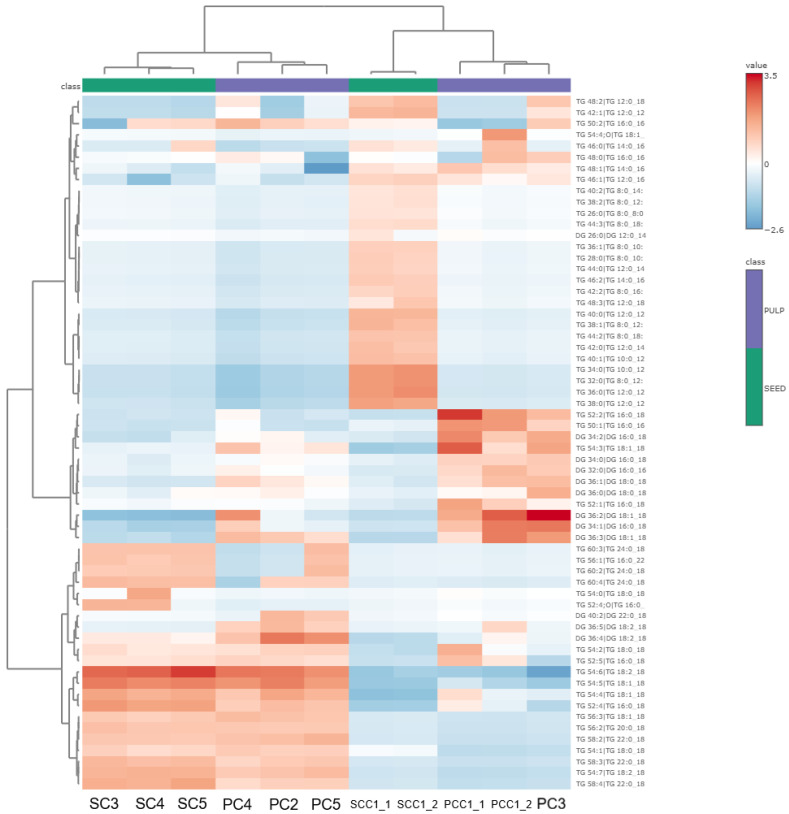
Heatmap of the lipid profile of different commercial macauba *(Acrocomia aculeata)* oil samples from pulp and seed (x-axis). The y-axis contains two lipid classes: triacylglycerol (TG) and diacylglycerol (DG).

The lipids analysis by LC-HRMS showed the presence of diglycerides (DGs), oxidized triglycerides (TGox), and triglycerides (TGs), with the latter being the major lipid class in commercial macauba pulp and seed oil samples. In the heatmap, it is possible to observe differences between the pulp and seed groups, as well as variability among samples within each group, primarily in the distribution of triglyceride classes (Figure 5).

Only three oxidized triglycerides (TGox) species were detected. TG 18:1_18:1_9:1; O was found only in PCC1_1, representing 0.03% of its relative percentage (Table S3). TG 16:0_18:2_18:2;O was present in most samples, PCC1_1 (0.07%), PCC1_2 (0.05%), PC2 (0.04%), PC3 (0.01%), PC4 (0.04%), PC5 (0.05%), SC3 (0.17%), SC4 (0.17%), and SC5 (0.15%). Additionally, TG 18:1_18:2_18:1; O was also detected in most samples, PCC1_1 (0.51%), PCC1_2 (0.23%), SCC1_1 (0.01%), PC2 (0.08%), PC3 (0.18%), PC4 (0.14%), PC5 (0.10%), SC3 (0.10%), SC4 (0.11%) and SC5 (0.11%). The presence of oxidized TGs, especially in PCC1_1, SC4, and SC3, indicates aging processes or inadequate storage conditions [34].

When comparing the pulp and seed groups, a distinct profile is evident for the SCCs and PCCs samples, while the others show more similarities. SCCs samples contain a greater number of TG species and exhibit a more heterogeneous TG profile. These samples are composed mostly of TGs with short and saturated chains (32–38 carbon atoms, primarily containing C12:0). In contrast, PCCs samples exhibit a more homogeneous profile, with long and unsaturated TG chains (52–54 carbon atoms, mainly composed of C18:1). The PC3 sample presented a profile more similar to PCCs samples, but with additional contributions from TG 50:2 and 48:2. PC2, PC4, and PC5 show more comparable profiles, as these samples were rich in TGs with longer carbon chains and with relevant contributions from TG 54:6 and 54:5, primarily containing C18:2. SC3, SC4, and SC5 samples display a profile closer to PCs samples but present notable amounts of TG 52:4, 54:7, and 58:4.

#### 2.3.2. Lipids Quantification

**Figure 6:**
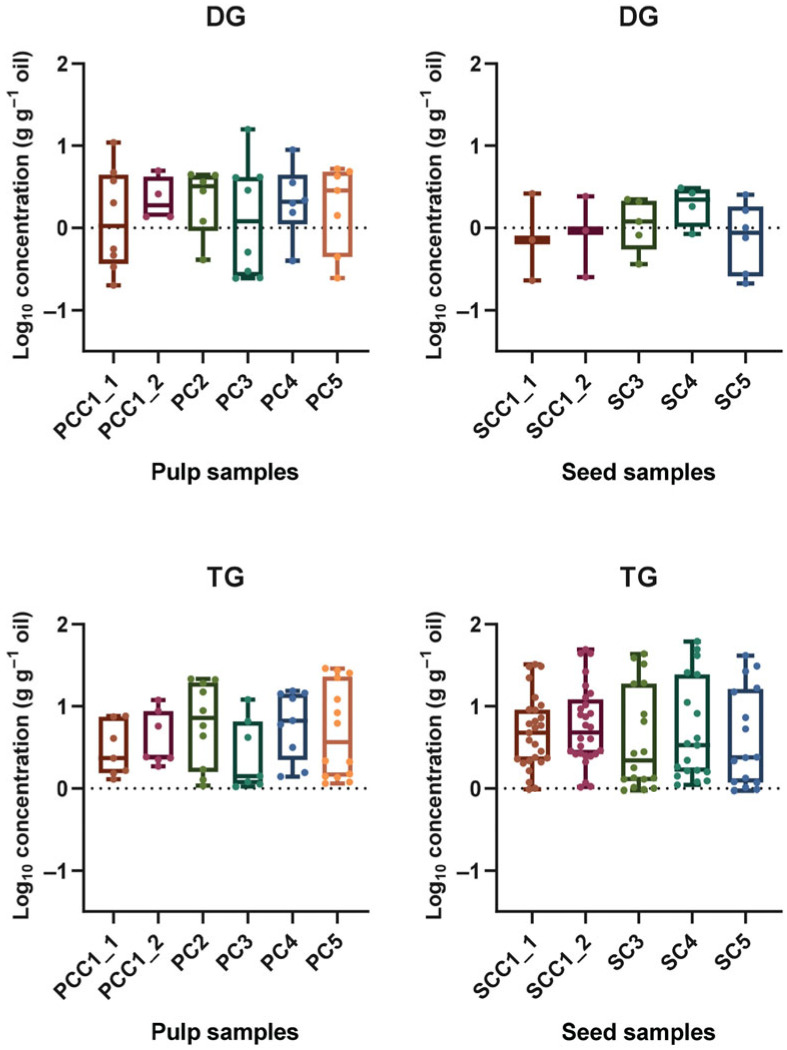
Box plot of the concentrations of lipids in commercial macauba (*Acrocomia aculeata*) pulp and seed oils, expressed on a log_10_ scale (g 100 g oil^−1^). The lipid classes represented are triacylglycerols (TGs) and diacylglycerols (DGs).

The results of the quantification of DGs and TGs in macauba seed and pulp oils are presented in Figure 6. The results are shown as total DG and TG contents. In total, 67 different lipid compounds were annotated, although approximately 37 of those were below the limit of quantification, as shown in Tables S3 and S4.

TGs were the most abundant class of lipids in macauba commercial seed and oil samples. Furthermore, these lipids exhibited a wide concentration range among the samples. Although DG concentrations varied less, pulp oils showed a broader distribution of these compounds when compared with seed oils. For TGs in commercial pulp oils, the concentration of total TGs ranged as follows: 1.29–7.59 g 100 g^−1^ oil for PCC1_1, 1.86–11.90 g 100 g^−1^ oil for PCC1_2, 1.07–21.62 g 100 g^−1^ oil for PC2, 1.05–12.11 g 100 g^−1^ oil for PC3, 1.39–15.38 g 100 g^−1^ oil for PC4, and 1.14–28.95 g 100 g^−1^ oil for PC5. Among these samples, PCC1_1, PCC1_2, PC3, and PC4 showed some degree of similarity, while PC2, PC4, and PC5 also formed a group with comparable ranges. For TGs in seed oils, the samples showed higher maximum values, smaller differences between samples, but broader overall ranges. The total amounts of TGs in the seed oil samples were as follows: 0.98–32.40 g 100 g^−1^ oil for SCC1_1, 1.04–49.20 g 100 g^−1^ oil for SCC1_2, 0.95–43.63 g 100 g^−1^ oil for SC3, 1.10–61.74 g 100 g^−1^ oil for SC4, and 0.94–41.68 g 100 g^−1^ oil for SC5.

Both pulp and seed commercial oil samples contained lower concentrations of DGs than TGs. The pulp oils showed higher DG levels and wider variation compared with the seed group. The ranges for total DGs in pulp oils were as follows: 0.20–19.91 g 100 g^−1^ oil for PCC1_1, 1.37–4.96 g 100 g^−1^ oil for PCC1_2, 0.41–4.45 g 100 g^−1^ oil for PC2, 0.24–15.84 g 100 g^−1^ oil for PC3, 0.40–8.93 g 100 g^−1^ oil for PC4, and 0.25–5.24 g 100 g^−1^ oil for PC5. In seed commercial oil samples, the concentration of total DGs were as follows: 0.23–2.61 g 100 g^−1^ oil for SCC1_1, 0.25–2.42 g 100 g^−1^ oil for SCC1_2, 0.36–2.21 g 100 g^−1^ oil for SC3, 0.84–3.05 g 100 g^−1^ oil for SC4, and 0.21–2.54 g 100 g^−1^ oil for SC5.

Comparing lipid concentrations between all samples, seed oils contained higher values and broader ranges. This can be explained by the number of species of lipids present in the macauba seed oil samples. As shown in the FAME analysis, seeds contained fatty acid methyl esters ranging from C8:0 to C18:2, which aligns with the lipid profile identified by LC-HRMS, predominantly containing carbon chains from C8:0 to C18:2. In contrast, pulp samples showed fatty acid methyl esters primarily ranging from C16:0 to C18:2, consistent with the lipid species identified, which were mostly composed of C16:0 to C18:2 chains.

#### 2.3.3. Statistical Analysis of Commercial Macauba Oils

**Figure 7:**
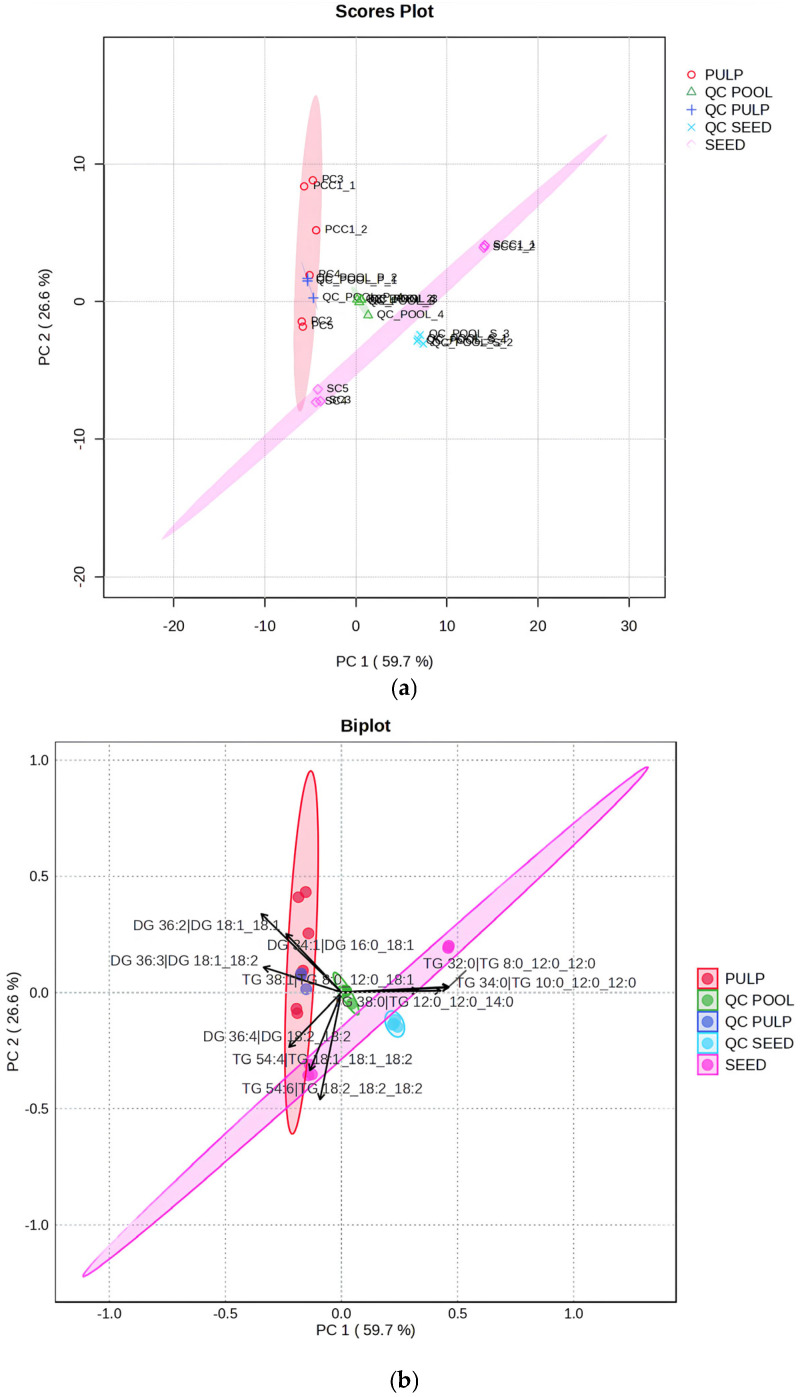
Scored (**a**) and biplot (**b**) plots of principal component analysis (PCA) of the lipid profile of commercial macauba (*Acrocomia aculeata*) oil samples from pulp and seed. The lipid classes listed in Figure (**b**) are triacylglycerol (TG) and diacylglycerol (DG).

Principal component analysis (PCA) was applied to evaluate the lipid profiles of the commercial samples, considering PCC1_1 and PCC1_2 as pulp control samples, as well as SCC1_1 and SCC1_2 as seed control samples. Figure 7a shows a clear differentiation between seed and pulp oil groups. Within the pulp group, PCC1_1, PCC1_2, and PC3 displayed profiles that were more distinct from PC2 and PC5, while PC4 was in the middle, showing similarities with both groups of pulp oil samples.

In the seed group, SCC1_1 and SCC1_2 showed similar profiles to each other, but showed a different profile from samples SC3, SC4, and SC5. These three samples were located in a region closer to the group of pulp oil samples, indicating chemical similarity between these samples.

The performance of the quality control (QC) samples further supports the reliability of the analysis. The global QC pool, composed of aliquots from all samples, was positioned near the center of the PCA score plot, indicating analytical stability and minimal instrumental drift. Likewise, the pulp QC pool clustered within the pulp sample group, and the seed QC pool clustered within the seed sample group. This behavior confirms good repeatability and demonstrates that the observed separations among samples are driven by genuine compositional differences rather than analytical variability. Furthermore, all annotated species showed a relative standard deviation below 30% between all pool QC samples.

Figure 7b highlights the most relevant lipids for each sample, and most of them are different from their corresponding control pulp and seed oil samples (PSCC and SSCC, respectively). Pulp samples PCC1_1, PCC1_2, and PC3 are primarily influenced by TG 54:3 (C18:1_18:1_18:1), DG 36:2, and DG 36:1, consistent with an oleic-rich profile. Seed samples SCC1_1 and SCC1_2 were strongly influenced by TG 36:0 (C12:0_12:0_12:0), TG 34:0 (C10:0_12:0_12:0), and TG 32:0 (C8:0_12:0_12:0), confirming their characteristic short/medium-chain lipid profile. The sample PC4 was strongly influenced by DG 36:3 (C18:1_C18:2). In contrast, samples PC2, PC5, SC3, SC4, and SC5 were heavily influenced by TG 54:6 (C18:2_18:2_18:2), DG 36:4 (18:2_18:2), and TG 54:4 (18:2_18:1_18:1). These lipids are key markers in distinguishing these samples from typical pulp and seed groups.

The opposite direction of vectors associated with SCC samples compared to those of PSCC and PC3 suggests negative correlations between their lipid compositions, reinforcing the separation of pulp and seed oils. Overall, although some minor lipids are common in both seed and pulp oils, the global profiles remain clearly distinct, as expected. Additionally, the clustering of QC samples demonstrates good analytical repeatability, low technical variability, and the robustness of the LC-HRMS method.

**Figure 8:**
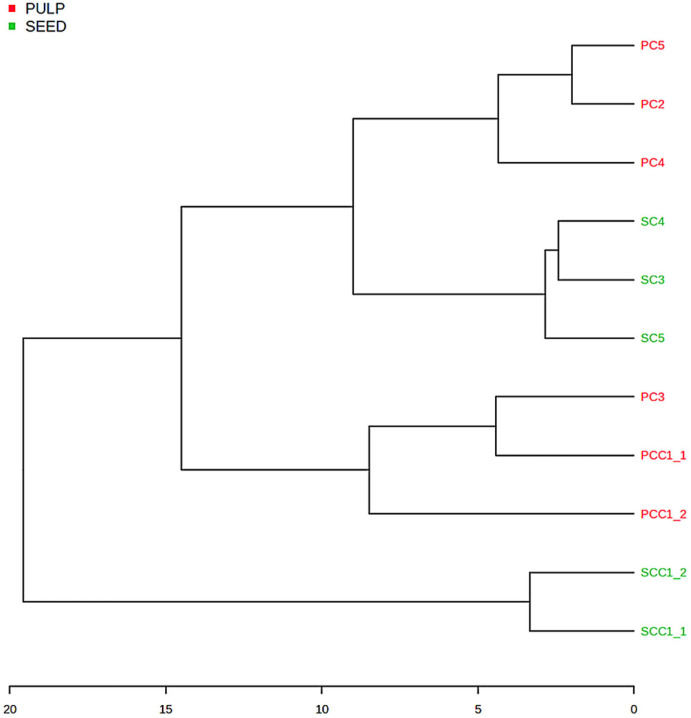
Dendrogram of the lipid profile of commercial macauba (*Acrocomia aculeata*) oil samples from pulp and seed.

Hierarchical clustering analysis was also performed as a complement to the PCA. The results are shown in Figure 8. A clear separation between pulp and seed oils can be observed, indicating significant compositional differences between the two types of oils. The seed control samples (SCC1_1 and SCC1_2) form a well-defined cluster that is clearly separated from the other groups, reflecting a characteristic and consistent lipid profile. The commercial seed samples (SC3, SC4, and SC5) form a coherent cluster, reflecting strong similarity among themselves. This cluster is positioned closer to the group of commercial pulp samples PC2, PC4, and PC5 than to the seed controls, suggesting partial convergence in lipid composition, potentially associated with shared triacylglycerol and diacylglycerol profiles.

Within the pulp group, two main subclusters are evident. PC2, PC4, and PC5 group together, indicating similar lipid compositions among these commercial pulp oils. In contrast, PCC1_1, PCC1_2, and PC3 form a separate and well-defined subcluster, reflecting a distinct pulp lipid profile and highlighting intra-group variability among pulp samples.

## 3. Discussion

It is important to highlight that this is the first study to analyze commercial macauba *(Acrocomia aculeata)* oils in scientific literature. Furthermore, it is also the first study to employ validated quantitative GC-FID and LC-HRMS methods for the analysis of macauba oils. The analysis of commercial macauba oils is important to ensure product quality. Lipid composition of oils is essential for determining the stability and suitability for food, cosmetics, and skincare products [25,35,36]. In the market, macauba pulp oil stands out for its nutritional profile and culinary potential, while the seed oil is more valued by the cosmetic and pharmaceutical industries due to its high lauric acid methyl ester content [18,19,20,25].

The FAME composition of the control samples reflects the intrinsic biological differences between the fruit tissues of *Acrocomia aculeata*. The pulp control samples (PCC1_1 and PCC1_2) are characterized by a methyl ester of oleic and palmitic acid profile, with linoleic acid in minor proportions, in agreement with profiles previously reported in the literature [7,8,9]. In the pulp, lipids act primarily as energy reserves that support fruit development and maturation, also contributing to fruit palatability and attractiveness to dispersers during the final stages of ripening [37,38].

In contrast, the seed control samples (SCC1_1 and SCC1_2) exhibit a distinct fatty acid methyl ester profile characterized by medium-chain saturated fatty acids, aligning with the known fatty acid profile of macauba seed oil [8,12,26]. Seed lipids function as dense energy reserves that sustain germination and early seedling growth [37,38,39]. The presence of saturated medium-chain fatty acid methyl esters such as C14:0, C12:0, C10:0, and C8:0 is typical of seed oils within the Arecaceae family and differs from the pulp profile, demonstrating clear tissue-specific lipid biosynthesis pathways [37].

Among the pulp oils, the control samples and the other commercial sample PC3 showed similar profiles, with a higher amount of C18:2, ranging from 23.64 to 39.10 g 100 g oil^−1^. The presence of small amounts of short- and medium-chain fatty acid methyl esters in pulp samples PC2, PC3, PC4, and PC5 may be explained using the same processing equipment, leading to cross-contamination.

Regarding the macauba seed oils, a wide variability was observed between the control and the other commercial samples. Samples SC3, SC4, and SC5 exhibited significantly different fatty acid methyl ester profiles, with elevated C18:2 contents ranging from 48.72 to 69.15 g 100 g oil^−1^. Additionally, in these samples, short- and medium-chain fatty acids (C8–C14), which are characteristic of macauba seed oil, were not detected.

The lipid analysis by LC-HRMS corroborates the FAME results. GC-FID and LC-HRMS were used in a complementary way to characterize macauba oils, allowing the quantification of FAMEs and the analysis of intact lipids such as DGs and TGs. The combination of these techniques provided a broad and consistent comprehension of the lipid composition of the samples. In control pulp samples, GC-FID showed C18:1 as the major fatty acid, while in the control seed samples, C12:0 was predominant. LC-HRMS supported these findings by showing the dominance of TG 54:3 (C18:1_18:1_18:1) in pulp oils and TG 36:0 (C12:0_12:0_12:0) in seed oils.

LC-HRMS also confirmed the discrepancies observed in several commercial samples that showed high methyl ester of linoleic acid (C18:2) levels in GC-FID analysis, revealing that the predominant lipid in these divergent samples was TG 54:6 (C18:2_18:2_18:2). The simultaneous use of GC-FID and LC-HRMS is justified by the complementary advantages of each technique. GC-FID provides robust quantification and allows broad comparison with the literature, though it requires derivatization and does not preserve structural information of intact lipids, whereas LC-HRMS enables the identification of intact and specific molecular lipid species without the need for volatilization. This expands the understanding of lipid structure and chemical composition. While previous studies reported lipids only as relative percentages, this work is the first to present quantitative data and to annotated 67 lipid species, including DG and TGox species. All the species annotated can be found in Table S3.

**Figure 9:**
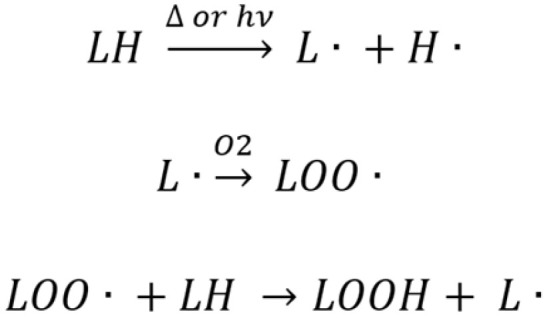
Triglyceride (TG) oxidation mechanisms.

TGox species appear in the samples in small amounts, but they are directly related to the overall quality of the oil. As shown here, commercial macauba oil samples are rich in unsaturated fatty acids, mainly C18:1 and C18:2, which increases their susceptibility to oxidation. Even though the oils in this study were extracted by pressing, this process can still generate localized heating. Combined with exposure to light and oxygen, as described in the mechanism in Figure 9, these conditions can lead to oxidative processes [34,40,41]. In addition, poor storage and packaging conditions can contribute to these reactions [34].

When oil is exposed to heat, light, or reactive oxygen species, a hydrogen is removed from the lipid molecule (LH), forming an alkyl radical (L^●^). This radical reacts with oxygen (O_2_) to produce a peroxyl radical (LOO^●^). Then, the peroxyl radical abstracts a hydrogen from another lipid molecule, initiating a chain oxidation reaction and forming lipid hydroperoxides (LOOH), which are primary oxidation products. These initial products can further decompose into a variety of secondary compounds, including aldehydes, ketones, and epoxides. These compounds are responsible for the deterioration of oil quality, leading to changes in nutritional value, flavor, and odor [34,40,42,43]. In this study, only three TGox species were detected. TG 45:3;O|TG 18:1_18:1_9:1;O. was found only in the PCC1_1 sample. TG 52:4;O|TG 16:0_18:2_18:2;O was detected in all commercial samples, with the highest levels in SC4, followed by SC3 and SC5, and lower levels in PC2, PCC1_1, and PC4. TG 54:4;O. Finally, TG 18:1_18:2_18:1;O was found in most samples, except SCC1_2, with higher concentrations in PCC1_1, SC4, SC3, SC5 and PC5. None of these species were found above the quantification limit.

In the annotated lipids table (Table S4), a considerable number of species were below the limit of quantification. Considering this, the use of commercial deuterated standards optimized for plasma, such as SPLASH™ LIPIDOMIX^®^, may introduce quantification inaccuracies to the analysis when applied to matrices with substantially different lipid levels. However, validation of the method helps to minimize this type of issue.

In the commercial oil samples (Table S4), the lipid profile varied according to the oil source. Pulp control, PC3 and PC4 samples were mainly characterized by oleic-rich species, with TG 18:1_18:1_18:1 quantified at 7.59–14.48 g·100 g oil^−1^ (PCC1_1, PCC1_2, PC3, and PC4) and TG 16:0_18:1_18:1 at 6.57–8.79 g·100 g oil^−1^ (PCC1_1, PCC1_2, and PC3). In addition, DG 18:1_18:1 was present at 10.91 g·100 g oil^−1^ in PCC1_1 and 15.84 g·100 g oil^−1^ in PC3, indicating a relevant contribution of diacylglycerols to the pulp oil composition.

In contrast, seed control samples showed a lauric profile dominated by medium-chain triacylglycerols, with TG 12:0_12:0_12:0 quantified at 32.40–49.20 g·100 g oil^−1^, TG 10:0_12:0_12:0 at 30.61–44.18 g·100 g oil^−1^ and TG 8:0_12:0_12:0 at 30.70–44.29 g·100 g oil^−1^ for SCC1_1 and SCC1_2. However, other commercial samples, pulp and seed (PC2, PC4, PC5, SC3, SC4, and SC5) exhibited a markedly different profile, with polyunsaturated species prevailing, particularly TG 18:2_18:2_18:2, which reached 21.62–61.74 g·100 g oil^−1^, along with TG 18:1_18:2_18:2 (21.19–49.69 g·100 g oil^−1^). To better visualize and discriminate these compositional differences, a PCA was applied using the quantified lipid species (Figure 7).

The multivariate statistical analysis provides support for the discrimination between pulp and seed oils based on their lipid profiles. The PCA scores plot (Figure 7a) shows a clear separation between macauba pulp and seed oils. The seed oil samples (SC3, SC4, and SC5) are distributed along the negative PC1 axis and cluster together, reflecting similar lipid compositions. The control seed oil samples (SCC1_1 and SCC1_2) cluster closely together in the positive PC1 axis and show strong mutual correlation, confirming their compositional consistency. However, their separation from SC3, SC4, and SC5 indicates significant differences between control and other commercial macauba seed oils, clearly reflecting the variations in samples. In contrast, pulp samples are positioned on the negative side of PC1, forming a distinct group with considerable dispersion along the PC2 axis, suggesting higher intra-group variability among commercial pulp oils.

The PCA biplot (Figure 7b) enhances the interpretation of these groupings by revealing correlations between samples and specific lipid classes. PC2, PC5, SC3, SC4, and SC5 are strongly associated with triacylglycerols (TGs) and diacylglycerols (DGs) predominantly composed of methyl ester of linoleic acid (C18:2), indicating that these molecular species are key drivers of the observed clustering. However, PCC1_1, PCC1_2, and PC3 display similar projection vectors, indicating highly correlated lipid profiles, but show weak correlation with PC2 and PC5. The PC4 sample is distinctly positioned and strongly associated with DG 36:3, composed mainly of C18:1 and C18:2, highlighting compositional heterogeneity within the pulp oil group.

These relationships are further corroborated by the hierarchical clustering analysis (Figure 8), which independently confirms the PCA results. The dendrogram shows a primary bifurcation separating pulp and seed control oils, reinforcing tissue origin as the dominant discriminant factor. Within each main branch, subclusters reflect compositional similarities and differences consistent with the PCA. Particularly, the pulp subgroup SC3, SC4, and SC5 highlight the composition distinct from the expected profile for macauba seed macauba oil samples. Instead, these samples cluster closely with the macauba pulp oils subgroup, specifically PC2, PC4, and PC5, sharing a high concentration of unsaturated long-chain fatty acid methyl esters.

The lipid profile of macauba oils is known to vary according to geographic origin, maturation stage, post-harvest handling, and storage conditions, and such variability has been consistently reported in the literature based on fatty acid methyl ester composition [10,44,45,46]. For pulp oil, methyl ester of linoleic acid (C18:2) contents have been described approximately 7.5% to 35%, depending on location and agronomic factors, which may explain the elevated C18:2 level observed in sample PC4, as this sample still presents a substantial proportion of oleic acid (C18:1), consistent with reported pulp oil profiles [10].

However, the higher C18:2 contents observed in samples PC2, PC5, SC3, SC4, and SC5 exceed the upper limits reported for macauba pulp oils. While cultivar-dependent variations in fatty acid composition have been described for macauba, available studies indicate that these differences do not result in methyl ester of linoleic acid levels substantially above 35% in pulp oils [10,44].

Similarly, cross-contamination between pulp and seed during oil extraction could partially affect FAME proportions. However, seed oils typically contain low levels of linoleic acid, generally below 10%, which cannot explain the high values observed in these samples [10,45,46]. In addition, seed oils are characterized by significant amounts of medium-chain fatty acid methyl esters, mainly C12:0, C10:0, and C8:0, which are consistently reported in the literature. Thus, the absence or negligible levels of these FAMEs in samples SC4 and SC5 are inconsistent with a pulp–seed mixing scenario.

Processing conditions, such as extraction method or thermal exposure, may influence minor lipid components or oxidative status, but they are not expected to selectively enrich C18:2 to levels beyond those dictated by the intrinsic FAME composition of the raw material [26]. Therefore, while natural biological variability, cultivar differences, and technological factors can explain moderate deviations, they do not fully justify the unusually high linoleic acid contents detected in some commercial samples of macauba oil. From an analytical standpoint, the prevalence of highly unsaturated triglycerides like TG 54:6 serves as a key biomarker for identifying a possible contamination, given the lipid differences observed in PC2, PC5, SC3, SC4, and SC5. However, these findings also carry significant technological weight. For the industry, an oil with unexpectedly high unsaturation will have lower oxidative stability and a shorter shelf-life, directly impacting its storage and processing requirements [47,48,49].

By providing this detailed molecular approach, this study moves beyond quantification; it promotes a guide for macauba seed and pulp oil quality control. Understanding the balance between the polyunsaturated and saturated TG components of the oils is essential for selecting the right application, whether it is the food industry seeking stable oils or the cosmetic sector requiring specific emollient properties [7,20,26,50]. This ensures that macauba oils are used based on their real chemical strengths, promoting more reliable and high-quality bio-based products.

## 4. Materials and Methods

### 4.1. Reagents and Materials

Acetonitrile, hexane, isopropanol, methanol, and methyl-*tert*-butyl ether (LC-MS grade) were obtained from BioGrade (Durham, NC, USA). Formic acid and ammonium formate (LC-MS grade) were purchased from Tedia (Fairfield, OH, USA). Ultrapure water with a resistivity of 18.2 M Ω cm^−1^ was generated by a Millipore Milli-Q water purification system (Billerica, MA, USA). The lipid mixture SPLASH™ LIPIDOMIX^®^ was purchased from Avanti Polar Lipids (Alabaster, AL, USA). The methyl esters mixture FAMEMix C14-C22, methyl *n*-heptadecanoate ≥ 99%, sodium hydroxide ≥ 97%, ammonium chloride ≥ 99%, and sulfuric acid ≥ 95% were acquired from Sigma Aldrich (St. Louis, MO, USA). 

Eleven samples of Brazilian commercial macauba oil obtained by cold pressing were analyzed in this study. Six samples were from macauba pulp oil, and five from seed oil. The samples were obtained from 5 different companies.

Some samples were selected as control samples because their profiles are fully consistent with the literature, and the producing region and harvest dates are well-known. These samples were obtained from the same company (company 1) The control samples for pulp oils are from two harvest years: from harvest 1 (PCC1_1) and from harvest 2 (PCC1_2). The control sample for the seed oils was also obtained from two harvest years, the same harvests from the pulp oil. These control samples are from harvest 1 (SCC1_1) and from harvest 2 (SCC1_2). These samples were sourced from the northern region of Minas Gerais, a Cerrado area. Harvest 1 is from 2022/2023, while harvest 2 is from 2024/2025.

The remaining commercial samples were obtained from different companies: PC2 is pulp oil from company 2; PC3 and SC3 are pulp and seed oils, respectively, from company 3; PC4 and SC4 are pulp and seed oils, respectively, from company 4; and PC5 and SC5 are pulp and seed oils, respectively, from company 5.

### 4.2. Methods Validation

The validation of the methods for LC-HRMS and GC-FID was performed according to the ANVISA guidelines (National Health Surveillance Agency, Brazil) and FDA guidelines (Food and Drug Administration, USA), and other literature sources [31,32,51,52]. The validation parameters evaluated were linearity, the limits of detection (LOD) and quantification (LOQ), accuracy and precision, selectivity, and carryover.

For FAME analysis, the internal standard employed to quantify fatty acid methyl ester (FAME) was the methyl *n*-heptadecanoate (C17:0), purchased from Sigma-Aldrich (St. Louis, MO, USA). It was chosen as the internal standard because it is absent from the matrix and shares similar characteristics and behavior with the target analytes, belonging to the same lipid class.

For comprehensive lipid analysis, the commercial mix of deuterated lipid standards, SPLASH™ LIPIDOMIX^®^, was employed to quantify lipids from the classes: diglycerides (DG), oxidized triglycerides (TGox), and triglycerides (TG). Deuterated standard compounds are absent from the matrix but show behavior similar to the analytes of interest.

### 4.3. Linearity

The FAME method was validated using the internal standard: methyl *n*-heptadecanoate (C17:0). The calibration curve was analyzed in triplicate in six different concentration levels.

The method for comprehensive lipid analysis by LC-HRMS was validated using the SPLASH™ LIPIDOMIX^®^ as internal standard. The calibration curve was analyzed in triplicate using eight different concentrations for DG 33:1(d7)|DG 15:0_18:1(d7) and TG 48:1(d7)|TG 15:0_18:1(d7)_15:0.

Both methods (GC-FID and LC-HRMS) were constructed to evaluate the linear range by linear regression, as well as the R-squared coefficient (R^2^). The homoscedasticity of the methods was evaluated by the Cochran test.

### 4.4. Limit of Detection (LOD) and Quantification (LOQ)

For GC-FID and LC-HRMS methods, the sensitivity of the analytical method was evaluated by determining the LOD and LOQ. These parameters were calculated based on the standard deviation of the response and the slope of the calibration curves, utilizing data derived from the regression analysis. The limits were obtained using Equations (1) and (2), where σ represents the standard error of the regression and S represents the slope of the calibration curve.

LOD = (3.3 × σ)/S(1)

LOQ = (10 × σ)/S(2)

### 4.5. Accuracy and Precision

For GC-FID and LC-HRMS methods, accuracy was calculated by dividing the average experimental concentration by the theoretical concentration and multiplying by 100. The expected value should be between 85 and 115%.

For the precision determination of the GC-FID method FAME, the internal standard (C17:0) was added in three concentrations (small, medium, and high) to the macauba FAME samples. The relative standard deviation (%RSD) was calculated for samples analyzed on the same day and on three consecutive days.

For comprehensive lipid analysis by LC-HRMS, the internal standard SPLASH™ LIPIDOMIX^®^ was added in the same range of three concentrations to the macauba oil samples. Intra-day precision was calculated from the relative standard deviation of replicates on the same day, while inter-day precision was calculated from the %RSD of replicates analyzed on three consecutive days.

### 4.6. Selectivity

For both methods, selectivity or matrix effect was evaluated by analyzing six blank samples before the macauba oil samples containing the internal standard in three concentrations (small, medium, and high). The lack of analytes present in the retention time of the internal standards was considered a lack of matrix effect.

### 4.7. Carryover

Carryover was established after evaluating solvent blanks injected after analyses of the highest calibration points with the standards. For the carryover to the GC-FID and comprehensive lipids analysis by LC-HRMS methods, the response in solvent blanks for their respective *m*/*z* to the analytes in IS (C17:0) and in SPLASH™ LIPIDOMIX^®^ (DG 33:1(d7)|DG 15:0_18:1(d7) and TG 48:1(d7)|TG 15:0_18:1(d7)_15:0) should be <20% of the response of LOQ.

### 4.8. GC-FID and GC-MS Analysis for FAME

The analysis of fatty acid methyl esters (FAMEs) was carried out according to the literature, with adaptations [53]. Qualitative analyses were performed using a gas chromatograph (Agilent 6890N; Agilent, Santa Clara, CA, USA) equipped with a flame ionization detector (GC-FID) and a J&W DB-Wax capillary column (30 m × 0.25 mm × 0.25 μm; Agilent, Santa Clara, CA, USA), operating with a split ratio of 1:50 and an injection volume of 1 µL. The oven temperature program consisted of an initial step at 100 °C for 3 min, followed by a ramp of 10 °C min^−1^ up to 240 °C, held for 5 min. Hydrogen was used as the carrier gas at a flow rate of 1.5 mL min^−1^. The injector and detector temperatures were set at 230 °C and 260 °C, respectively.

Identification of FAMEs was performed by comparing retention times with a commercial standard mixture (C14–C22 FAME; Supelco, Bellefont, PA, USA). Confirmation of compounds present in the standard was conducted using a gas chromatograph (Agilent 7820A; Agilent, Santa Clara, CA, USA) coupled to a quadrupole mass spectrometer (GC–MS, Agilent 5977E; Agilent, Santa Clara, CA, USA), under the same chromatographic conditions, with the mass spectra compared to those obtained from the FAME standard mixture (C14–C22).

### 4.9. FAME Quantification in Macauba Oil by GC

For the quantification of fatty acids, derivatization into fatty acid methyl esters (FAMEs) was performed according to the literature with adaptations [54,55]. For the preparation of the esterification solution, 2 g of ammonium chloride is added to 60 mL of methanol, followed by 3 mL of concentrated sulfuric acid in refluxed for 15 min.

Samples of macauba oils (100 mg) were weighed into 50 mL Falcon tubes. Next, 1.5 mL of 0.5 M NaOH solution in methanol was added, and the mixture was heated at 60 °C for 5 min under constant stirring. Subsequently, 4.5 mL of the esterification solution was added, and the reaction was maintained for an additional 5 min under the same conditions.

After cooling, 10 mL of hexane and 10 mL of distilled water were added, followed by homogenization using a vortex mixer. The mixture was centrifuged at 5000 rpm for 10 min, the organic phase was collected, and then the solvent was evaporated under a nitrogen flow. For analysis, 10 µL of the oil obtained and 1450 µL of hexane LC grade were transferred to 2 mL vials containing 50 µL of methyl *n*-heptadecanoate solution in hexane (5 mg mL^−1^) as an internal standard.

### 4.10. Comprehensive Lipids by LC-HRMS

The LC-HRMS analysis was performed using a method developed by our research group, previously described in the literature [51,52]. The method was performed using a Dionex UltiMate 3000 liquid chromatography (Thermo Fischer Scientific, Waltham, MA, USA) coupled to a hybrid Quadrupole-Orbitrap high-resolution mass spectrometer (Thermo Q-Exactive Plus; Thermo Fischer Scientific, Waltham, MA, USA) equipped with an electrospray ionization (ESI) source. Chromatography separation was performed in a Waters XSelect CSH C18 column (150 mm × 2.1 mm; 2.5 μm particle size; Waters, Milford, MA, USA) in gradient elution mode using acetonitrile/water (60:40, *v*/*v*) as mobile phase A and isopropanol/acetonitrile (90:10, *v*/*v*) as mobile phase B, both with 0.1% formic acid and 10 mM ammonium formate. The gradient elution was as follows: 0–2.0 min 40% B; 2.0–2.1 min 43% B; 2.1–12.0 min 50% B; 12.0–12.1 min 54% B; 12.1–18.0 min 70% B; 18.0–18.1 min 99% B; 18.1–25.0 min 40% B. The column temperature was set to 45 °C and the solvent flow rate was 0.4 mL min^−1^. Sample injection was 5 μL for both positive and negative ESI modes. Data were acquired in full scan over an *m*/*z* range of 100–1000 Da at a resolution of 35.000 (FWHM), followed by a ddMS2 Top 5 experiment at a resolution of 17.500. Quality control samples (pooled QC, seed pooled QC, and pulp pooled QC) were prepared by mixing all the samples (for pooled QC), seeds, and pulp samples (for seed and pulp pooled QC, respectively) in equal amounts into the same vial and analyzed every 5 samples.

### 4.11. Lipid Quantification in Macauba Oil

The macauba oil samples were diluted to 2.75 mg mL^−1^ in isopropanol. Then, 1 μL of sample was added to 47 μL of isopropanol, and 7 μL of internal standard (SPLASH™ LIPIDOMIX^®^) was added [56]. The final concentration was 50 μg. mL^−1^ of macauba oils, and for the internal standard was 7 μg mL^−1^ of TG 15:0–18:1–15:0 (2d7) and 1.27 μg mL^−1^ of DG 15:0–18:1 (2d7). Afterwards, the samples were subjected to chromatographic analysis.

### 4.12. Data Processing

The FAME data obtained from GC-MS were analyzed by Agilent MSD ChemStation E.02.02.1431 software, and the compounds’ annotation was performed using the NIST Mass Spectral library v14.0. The data from the GC-FID was quantified by the Agilent ChemStation B.04.01 software.

The lipid data obtained from LC-HRMS were analyzed by the software Thermo Xcalibur v2.2 and processed by the software MS-DIAL v4.00 [57]. The processing parameters include peak detection, deconvolution, alignment, and compound annotation using a default lipid MS/MS library, all described in Table S1 in the Supplementary Materials. From the MS-DIAL software, a list of identified lipids was exported as a CSV file and used in the construction of a database used in the quantification of the lipids in TraceFinder v4.0 software (Thermo Scientific Fischer Scientific, Waltham, MA, USA). All lipids were annotated using a mass error below 5 ppm for the *m/z* values of the adducts of interest. Characteristic MS/MS fragments from the lipid classes were observed with mass error below 10 ppm; all the parameters used are described in Table S2 [51].

### 4.13. Statistical Analysis

Statistical analyses from data obtained for FAME analysis and LC-HRMS lipid analysis were performed using the software GraphPad Prism v8.0 and Python v3.14.2. The data processed and exported from the MS-DIAL were processed for multivariate analysis in the Metaboanalyst 6.0 web server (http://www.metaboanalyst.ca; accessed on 4 December 2025) [58]. The parameters used were normalization by constant sum, square root transformation, and Pareto Scaling. The statistical tests were performed at a significance level of 5%.

## 5. Conclusions

This study provides the first detailed quantitative analysis of commercial macauba *(Acrocomia aculeata)* oils in Brazil by LC-HRMS and GC-FID/GC-MS. The validated methods showed high precision and accuracy. The analyses confirmed a clear chemical distinction between pulp and seed macauba oils, both composed only of diglycerides (DGs), oxidized triglycerides (TGox), and triglycerides (TGs). Commercial samples showed strong variability. Some pulp (PCC1_1, PCC1_2, PC3) and seed (SCC1_1, SCC1_2) oils matched expected profiles. For the pulp oils, oleic acid (C18:1) was predominant, while seed oil displayed a lauric-rich profile (C12:0), including other short-chain fatty acids. However, the sample PC4 showed a more distinct profile, with more C18:2 and C18:1; this discrepancy can be related to variations in the harvest location of the macauba fruit and storage conditions, for instance. Furthermore, several samples (PC2, PC5, SC3, SC4, SC5) diverged and had unusually high levels of linoleic acid (C18:2). Lipid analysis also showed TG 54:6 as the main species in these atypical samples. The PCA confirmed the separation of these samples from typical pulp and seed oil samples. Overall, the lipid profile, especially TG distribution, proved to be a useful biomarker for identifying possible contamination in commercial macauba pulp and seed oils. This study also offers important, detailed data on FAMEs and lipid species to support quality control and industrial applications of macauba oils.

## Figures and Tables

**Table 1:** Validation parameters evaluated for the quantification of fatty acid methyl esters using GC-FID (GC) and for the lipid quantification using LC-HRMS (LC) in macauba (*Acrocomia aculeata*) pulp and seed oil obtained by cold pressing.

Method	Standard	t_R(min)_	R^2^	LOD (mg mL^−1^)	LOQ (mg mL^−1^)	Linear Range (mg mL^−1^)	Intra-Day Precision	Inter-Day Precision	Accuracy (%)	Carryover/Matrix Effect	Equity of Variance
GC	methyl *n*-heptadecanoate	13.54	0.99	0.0637	0.193	0.1–6.0	0.80	2.59	97	Absent	Homoscedastic
LC	DG 33:1(d7)	15.46	0.99	0.0343	0.1039	0.1–2.7	4.17	24.59	85.84	Absent	Homoscedastic
	TG 48:1(d7)	18.08	0.99	0.1558	0.4722	1.0–13.0	3.81	8.92	86.97	Absent	Homoscedastic

t_R(min)_: retention time (minutes); R^2^: correlation coefficient; LOD: limit of detection; LOQ: limit of quantification; intra-day precision: relative standard deviation (RSD%) for triplicates of samples analyzed on the same day in three different concentration levels; inter-day precision: relative standard deviations (RSD%) for triplicates of samples analyzed on three different days in three different concentrations.

**Table 2:** Fatty acid methyl esters (FAMEs) of macauba (*Acrocomia aculeata*) pulp and seed oil as percentage content.

FAME	Pulp Oil	Seed Oil
Caprylic acid methyl ester (C8:0)	-	0.84 ± 0.22
Capric acid methyl ester (C10:0)	-	2.69 ± 0.08
Lauric acid methyl ester (C12:0)	-	33.55 ± 1.30
Myristic acid methyl ester (C14:0)	-	9.79 ± 0.62
Palmitic acid methyl ester (C16:0)	20.07 ± 0.05	9.50 ± 0.31
Palmitoleic acid methyl ester (C16:1)	3.58 ± 0.04	-
Stearic acid methyl ester (C18:0)	2.76 ± 0.05	3.42 ± 0.24
Oleic acid methyl ester (C18:1)	59.19 ± 0.14	36.02 ± 2.27
Linoleic acid methyl ester (C18:2)	13.57 ± 0.03	4.19 ± 0.17
Linolenic acid methyl ester (C18:3)	0.83 ± 0.01	-

Results were expressed as mean ± standard deviation (*n* = 3); Dash (-): not detected.

**Table 3:** Concentration of fatty acid methyl esters (FAMEs) of commercial macauba (*Acrocomia aculeata*) pulp oils as g 100 g oil^−1^.

FAME	PCC1_1	PCC1_2	PC2	PC3	PC4	PC5
Caprylic acid methyl ester (C8:0)	-	-	-	-	-	-
Capric acid methyl ester (C10:0)	-	-	-	-	-	-
Lauric acid methyl ester (C12:0)	-	-	n.q.	n.q.	n.q.	n.q.
Myristic acid methyl ester (C14:0)	-	-	-	n.q.	-	-
Palmitic acid methyl ester (C16:0)	12.99 ± 0.58 ^a^	11.59 ± 0.58 ^b^	6.74 ± 0.10 ^c^	9.95 ± 0.09 ^d^	9.22 ± 6.47 ^d^	6.28 ± 0.39 ^c^
Palmitoleic acid methyl ester (C16:1)	2.33 ± 0.11 ^a^	2.29 ± 0.18 ^a^	n.q.	1.73 ± 0.01 ^b^	n.q.	n.q.
Stearic acid methyl ester (C18:0)	1.73 ± 0.10 ^a^	1.91 ± 0.08 ^a^	3.84 ± 0.03 ^b^	n.q.	2.80 ± 3.70 ^d^	3.59 ± 0.27 ^b^
Oleic acid methyl ester (C18:1)	38.37 ± 1.55 ^a^	52.76 ± 4.10 ^b,d^	30.68 ± 0.25 ^c^	56.30 ± 0.71 ^b^	49.15 ± 29.60 ^d^	28.73 ± 2.27 ^e^
Linoleic acid methyl ester (C18:2)	8.80 ± 0.36 ^a^	8.08 ± 0.35 ^a^	39.10 ± 0.36 ^b^	7.66 ± 0.09 ^a^	23.64 ± 37.65 ^c^	36.51 ± 2.78 ^b^
Linolenic acid methyl ester (C18:3)	n.q.	n.q.	n.q.	n.q.	n.q.	n.q.
Saturated fatty acids (SFAs)	14.72 ± 0.59	13.50 ± 0.58	10.58 ± 0.11	9.95 ± 0.09	12.02 ± 7.45	9.87 ± 0.47
Unsaturated fatty acids (UFAs)	49.49 ± 1.60	63.13 ± 4.12	69.78 ± 0.44	65.70 ± 0.71	72.79 ± 47.89	65.24 ± 3.59
Total content	64.21 ± 1.70	76.63 ± 4.16	80.36 ± 0.45	75.64 ± 0.72	84.81 ± 48.47	75.11 ± 3.62

Results were expressed as mean ± standard deviation (*n* = 3); Dash (-): not detected; n.q.: detected but not quantified; Values in the same line with different superscripts are significantly different (*p* < 0.05).

**Table 4:** Concentration of fatty acid methyl esters (FAMEs) of commercial macauba (*Acrocomia aculeata*) seed oils as g 100 g oil^−1^.

FAME	SCC1_1	SCC1_2	SC3	SC4	SC5
Caprylic acid methyl ester (C8:0)	n.q.	1.81 ± 0.89	-	-	-
Capric acid methyl ester (C10:0)	2.63 ± 0.15 ^a^	2.82 ± 0.53 ^a^	-	-	-
Lauric acid methyl ester (C12:0)	33.03 ± 2.24 ^a^	35.02 ± 4.34 ^a^	-	-	-
Myristic acid methyl ester (C14:0)	9.53 ± 0.72 ^a^	8.51 ± 0.96 ^a^	-	-	-
Palmitic acid methyl ester (C16:0)	8.88 ± 0.30 ^a^	7.53 ± 0.39 ^a,b^	6.19 ± 0.41 ^b^	7.72 ± 1.12 ^a,b^	6.44 ± 0.45 ^b^
Palmitoleic acid methyl ester (C16:1)	-	-	n.q.	-	-
Stearic acid methyl ester (C18:0)	3.26 ± 0.24 ^a^	2.45 ± 0.06 ^b^	4.22 ± 0.19 ^c^	4.57 ± 0.16 ^c^	4.64 ± 0.42 ^c^
Oleic acid methyl ester (C18:1)	33.56 ± 1.72 ^a^	31.77 ± 0.87 ^a^	24.05 ± 1.00 ^b^	29.41 ± 7.32 ^a,b^	23.11 ± 2.72 ^b^
Linoleic acid methyl ester (C18:2)	3.80 ± 0.29 ^a^	4.18 ± 0.10 ^a^	48.72 ± 2.22 ^c^	69.65 ± 10.28 ^b^	52.89 ± 4.83 ^c^
Linolenic acid methyl ester (C18:3)	-	-	n.q.	n.q.	n.q.
Saturated fatty acids (SFAs)	58.12 ± 2.40	58.13 ± 4.59	10.42 ± 0.45	12.29 ± 1.13	11.09 ± 0.62
Unsaturated fatty acids (UFAs)	37.36 ± 1.74	35.96 ± 0.87	72.77 ± 2.43	99.06 ± 12.62	76.00 ± 5.54
Total content	95.48 ± 2.97	94.08 ± 4.67	83.18 ± 2.48	111.36 ± 12.67	87.08 ± 5.58

Results were expressed as mean ± standard deviation (*n* = 3); Dash (-): detected but not detected; n.q.: not quantified; Values in the same line with different superscripts are significantly different (*p* < 0.05).

## Data Availability

The original contributions presented in this study are included in the article/Supplementary Materials. Further inquiries can be directed to the corresponding author.

## Supplementary Materials

The following supporting information can be downloaded at https://www.mdpi.com/article/10.3390/plants15020268/s1. Section S1: Chromatogram of FAME profile of macauba pulp and seed oil control samples; Section S2: MS Dial parameters; Section S3: Trace Finder parameters; Section S4: Total ion chromatogram (TIC) of the lipid profile of quality control sample (QC POOL 3); Section S5: Annotated compounds in the macauba oils commercial samples; Figure S1: Chromatogram of the FAME profile obtained through GC-FID of macauba pul (a) and seed (b) oil control sample; Figure S2: Total ion chromatogram (TIC) of the lipid profile of a quality control sample obtained through LC-HRMS on positive ion mode (a) and negative ion mode (b); Table S1: Parameters used in the processing of data obtained by LC-HRMS in the MS-DIAL 4.7 software; Table S2: Parameters used in the processing of data obtained by LC-HRMS in the TraceFinder 4.0 software; Table S3: Relative composition (%) of lipids species annotated in macauba commercial oils by LC-HRMS analysis; Table S4: Concentration in g 100g^−1^ macauba oils of annotated lipid species obtained by LC-HRMS analysis.

## Abbreviations

The following abbreviations are used in this manuscript:

ANVISA	National Health Surveillance Agency
DG	Diglyceride
ESI	Electrospray ionization
FAME	Fatty acid methyl ester
FDA	Food and drug administration
FFA	Free fatty acid
FID	Flame ionization detector
FWHM	Full width at half Maximum
GC	Gas chromatography
HRMS	High-resolution mass spectrometry
IS	Internal standard
LC	Liquid chromatography
LOD	Limits of detection
LOQ	Limits of quantification
MS	Mass spectrometry
PCA	Principal component analysis
QC	Quality control
RSD	Relative standard deviation
TG	Triglyceride
TGox	Oxidized triglyceride

## References

[1] Borges C.E., dos Santos J.C.B., Evaristo A.B., da Cunha T.G., Von dos Santos Veloso R., Barroso G.M., Souza P.G.C., da Silva R.S. (2021). Distribution and Future Projection of Potential Cultivation Areas for *Acrocomia aculeata* (Arecaceae) Worldwide: The Emerging Energy Culture of the Tropics. Theor. Appl. Climatol..

[2] Nobre D.A., Trogello E., Borghetti R.A., de Souza David A.M. (2014). Macaúba: Palmeira de Extração Sustentável Para Biocombustível. Colloq. Agrar..

[3] Sehnen C., Corrêa T.R., Grossi J.A., Castricini A., Ribeiro A.S. (2011). Exploração Sustentável Da Macaúba Para Produção de Biodiesel Colheita, Pós-Colheita e Qualidade Dos Frutos. Inf. Demogr..

[4] Madeira D.D.C., Motoike S.Y., Simiqueli G.F., Kuki K.N., de Melo Goulart S., Rigolon T.C.B., Nogueira P.T.S., da Silva Castro A., de Oliveira Couto E.G. (2024). Phenotypic Characterization and Genetic Diversity of Macauba (*Acrocomia aculeata*) Accessions Based on Oil Attributes and Fruit Biometrics. Genet. Resour. Crop Evol..

[5] Fernández-Coppel I.A., Barbosa-Evaristo A., Corrêa-Guimarães A., Martín-Gil J., Navas-Gracia L.M., Martín-Ramos P. (2018). Life Cycle Analysis of Macauba Palm Cultivation: A Promising Crop for Biofuel Production. Ind. Crops Prod..

[6] Sorita G.D., Favaro S.P., Gambetta R., Ambrosi A., Di Luccio M. (2025). Macauba (*Acrocomia* ssp.) Fruits: A Comprehensive Review of Nutritional and Phytochemical Profiles, Health Benefits, and Sustainable Oil Production. Compr. Rev. Food Sci. Food Saf..

[7] Sant’ Ana C.T., Do Carmo M.A.V., Azevedo L., Costa N.M.B., Martino H.S.D., de Barros F.A.R. (2025). Macauba (*Acrocomia aculeata*) Pulp and Kernel Oils, and Co-Products: Chemical Characterization and Antioxidant Properties. Cienc. Rural..

[8] Coimbra M.C., Jorge N. (2012). Fatty Acids and Bioactive Compounds of the Pulps and Kernels of Brazilian Palm Species, Guariroba (*Syagrus oleraces*), Jerivá (*Syagrus romanzoffiana*) and Macaúba (*Acrocomia aculeata*). J. Sci. Food Agric..

[9] Nunes A.A., Favaro S.P., Galvani F., Miranda C.H.B. (2015). Good Practices of Harvest and Processing Provide High Quality Macauba Pulp Oil. Eur. J. Lipid Sci. Technol..

[10] Antoniassi R., Cordeiro De Freitas S., Santos Silva T.D., De Araujo Santiago M.C.P., Wilhelm A.E., Vilela Junqueira N.T. (2020). Impact of Genotype on Fatty Acid Profile, Oil Content and Nutritional Value of the Sweet Fruits of *Acrocomia aculeate*. Rev. Bras. Frutic..

[11] de Oliveira Gonçalves T., Filbido G.S., de Oliveira Pinheiro A.P., Pinto Piereti P.D., Dalla Villa R., de Oliveira A.P. (2020). In Vitro Bioaccessibility of the Cu, Fe, Mn and Zn in the Baru Almond and Bocaiúva Pulp and, Macronutrients Characterization. J. Food Compos. Anal..

[12] Souza G.K., Diório A., Johann G., Gomes M.C.S., Pomini A.M., Arroyo P.A., Pereira N.C. (2019). Assessment of the Physicochemical Properties and Oxidative Stability of Kernel Fruit Oil from the *Acrocomia totai* Palm Tree. J. Am. Oil Chem. Soc..

[13] Silva R.B., Silva-Júnior E.V., Rodrigues L.C., Andrade L.H.C., Da Silva S.I., Harand W., Oliveira A.F.M. (2015). A Comparative Study of Nutritional Composition and Potential Use of Some Underutilized Tropical Fruits of Arecaceae. An. Acad. Bras. Cienc..

[14] Landmann W., Frampton V.L. (1968). Fatty Acid Composition of Mbocayá Palm (*Acrocomia totai*) Kernel and Pulp Oils. J. Am. Oil Chem. Soc..

[15] Teles H.D.F., Pires L.L., Garcia J., Rosa J.Q.S., Farias J.G., Naves R.V. (2011). Ambientes de Ocorrência Natural de Macaúba. Pesqui. Agropecu. Trop..

[16] da Costa Lima Pires P., da Silva César A., Cardoso A.N., Favaro S.P., Conejero M.A. (2023). Strategies to Improve the Competitiveness of an Agroindustrial System for a Macauba Based Oil Production in Minas Gerais State, Brazil. Land Use Policy.

[17] Cardoso A., Laviola B.G., Santos G.S., de Sousa H.U., de Oliveira H.B., Veras L.C., Ciannella R., Favaro S.P. (2017). Opportunities and Challenges for Sustainable Production of A. Aculeata through Agroforestry Systems. Ind. Crops Prod..

[18] César A.D.S., Almeida F.D.A., De Souza R.P., Silva G.C., Atabani A.E. (2015). The Prospects of Using *Acrocomia aculeata* (Macaúba) a Non-Edible Biodiesel Feedstock in Brazil. Renew. Sustain. Energy Rev..

[19] Aires G.C.M., de Carvalho Junior R.N. (2023). Potential of Supercritical *Acrocomia aculeata* Oil and Its Technology Trends. Appl. Sci..

[20] García Cabrera O., Magalhães Grimaldi L., Grimaldi R., Paula Badan Ribeiro A. (2023). Macauba (*Acrocomia aculeata*): Biology, Oil Processing, and Technological Potential. Oilseed Crops—Uses, Biology and Production.

[21] Lorenzi G.M.A.C., Negrelle R.R.B. (2006). *Acrocomia aculeata* (Jacq.) Lodd. ex Mart.: Aspectos Ecológicos. Visão Acad..

[22] Figueiredo A.L., Silva M.C., Pizzo J.S., Santos P.D.S., Manin L.P., Leôncio M.S., Visentainer J.V., Santos O.O. (2023). Evaluation of Lipid Composition and Nutritional Quality of Olive Oil Varieties Using ESI-MS, GC-FID and Chemometrics Techniques. J. Braz. Chem. Soc..

[23] Costa G.L.A., Buccini D.F., Arruda A.L.A., Favaro S.P., Moreno S.E. (2020). Phytochemical Profile, Anti-Inflammatory, Antimutagenic and Antioxidant Properties *Acrocomia aculeata* (Jacq.) Lodd. Pulp Oil. Food Sci. Technol..

[24] Da Silva L.P.R., Rodrigues E.L., Hiane P.A., Nunes Â.A., Filiú W.F., Cavalheiro L.F., Nazário C.E.D., Asato M.A., Freitas K.D.C., Bogo D. (2023). Bocaiuva (*Acrocomia aculeata*) Nut Oil: Composition and Metabolic Impact in an Experimental Study. Food Sci. Technol..

[25] Prates-Valério P., Celayeta J.M.F., Cren E.C. (2019). Quality Parameters of Mechanically Extracted Edible Macauba Oils (*Acrocomia aculeata*) for Potential Food and Alternative Industrial Feedstock Application. Eur. J. Lipid Sci. Technol..

[26] Del Río J.C., Evaristo A.B., Marques G., Martín-Ramos P., Martín-Gil J., Gutiérrez A. (2016). Chemical Composition and Thermal Behavior of the Pulp and Kernel Oils from Macauba Palm (*Acrocomia aculeata*) Fruit. Ind. Crops Prod..

[27] Lieb V.M., Schex R., Esquivel P., Jiménez V.M., Schmarr H.G., Carle R., Steingass C.B. (2019). Fatty Acids and Triacylglycerols in the Mesocarp and Kernel Oils of Maturing Costa Rican *Acrocomia aculeata* Fruits. NFS J..

[28] Dodds E.D., McCoy M.R., Rea L.D., Kennish J.M. (2005). Gas Chromatographic Quantification of Fatty Acid Methyl Esters: Flame Ionization Detection vs. Electron Impact Mass Spectrometry. Lipids.

[29] Tietel Z., Hammann S., Meckelmann S.W., Ziv C., Pauling J.K., Wölk M., Würf V., Alves E., Neves B., Domingues M.R. (2023). An Overview of Food Lipids toward Food Lipidomics. Compr. Rev. Food Sci. Food Saf..

[30] Murphy R.C., Axelsen P.H. (2011). Mass Spectrometric Analysis of Long-Chain Lipids. Mass Spectrom. Rev..

[31] ANVISA (2017). Ministério Da Saúde Agência Nacional de Vigilância Sanitária RESOLUÇÃO DA DIRETORIA COLEGIADA-RDC No 166, DE 24 DE JULHO DE 2017.

[32] FDA, CDER (2018). Bioanalytical Method Validation Guidance for Industry Biopharmaceutics Bioanalytical Method Validation Guidance for Industry Biopharmaceutics Contains Nonbinding Recommendations.

[33] de Souza F.I.L., Lima K.C., de Lima W.S., Mendes Oliveira R.M. (2024). Evaluation of Macaúba Oil: Extractive Yield, Quality, Nutritional Indexes, and Biodiesel Lipid Profile. Rev. Virtual Quim..

[34] Kato S., Shimizu N., Hanzawa Y., Otoki Y., Ito J., Kimura F., Takekoshi S., Sakaino M., Sano T., Eitsuka T. (2018). Determination of Triacylglycerol Oxidation Mechanisms in Canola Oil Using Liquid Chromatography–Tandem Mass Spectrometry. npj Sci. Food.

[35] Kamal-Eldin A. (2006). Effect of Fatty Acids and Tocopherols on the Oxidative Stability of Vegetable Oils. Eur. J. Lipid Sci. Technol..

[36] Pimenta T.V., Cano Andrade M.H., Antoniassi R. Extração, Neutralização e Caracterização dos Óleos do Fruto da Macaúba (*Acrocomia aculeata*). Proceedings of the XIX Congresso Brasileiro de Engenharia Química.

[37] Guerin C., Serret J., Montúfar R., Vaissayre V., Bastos-Siqueira A., Durand-Gasselin T., Tregear J., Morcillo F., Dussert S. (2020). Palm Seed and Fruit Lipid Composition: Phylogenetic and Ecological Perspectives. Ann. Bot..

[38] Montoya S.G., Motoike S.Y., Kuki K.N., Couto A.D. (2016). Fruit Development, Growth, and Stored Reserves in Macauba Palm (*Acrocomia aculeata*), an Alternative Bioenergy Crop. Planta.

[39] Afifi E.H., John Martin J.J., Wang Q., Li X., Liu X., Zhou L., Li R., Fu D., Li Q., Ye J. (2025). Fatty Acid and Lipid Metabolism in Oil Palm: From Biochemistry to Molecular Mechanisms. Int. J. Mol. Sci..

[40] Gomes T., Caponio F., Delcuratolo D. (2003). Fate of Oxidized Triglycerides during Refining of Seed Oils. J. Agric. Food Chem..

[41] Crossley A., Heyes T.D., Hudson B.J.F. (1962). The Effect of Heat on Pure Triglycerides. J. Am. Oil Chem. Soc..

[42] Yoshida Y., Umeno A., Shichiri M. (2013). Lipid Peroxidation Biomarkers for Evaluating Oxidative Stress and Assessing Antioxidant Capacity in Vivo. J. Clin. Biochem. Nutr..

[43] Zeng W., Liu X., Chao Y., Wu Y., Qiu S., Lin B., Liu R., Tang R., Wu S., Xiao Z. (2024). The Effect of Extraction Methods on the Components and Quality of Camellia Oleifera Oil: Focusing on the Flavor and Lipidomics. Food Chem..

[44] Oliveira C.D., Pereira e Silveira B.M., Fernanda de Assis N., Rios G.R., Siqueira-Silva A.I., Baffa Júnior J.C., Viana P.A., Pereira E.G. (2022). Synchronization between Photosynthetic Responses to Seasonality during Fruit Development and Fatty Acid Profile of Mesocarp Oil in Macauba (*Acrocomia aculeata*). Biocatal. Agric. Biotechnol..

[45] Barreto L.C., Magalhães A.L.L., Takahashi J.A., Garcia Q.S. (2016). Dynamic of Reserve Compounds of Mesocarp and Seeds of Macaw Palm (*Acrocomia aculeata*) Submitted to Different Storage Conditions. Trees-Struct. Funct..

[46] Alfaro-Solís J.D., Montoya-Arroyo A., Jiménez V.M., Arnáez-Serrano E., Pérez J., Vetter W., Frank J., Lewandowski I. (2020). *Acrocomia aculeata* Fruits from Three Regions in Costa Rica: An Assessment of Biometric Parameters, Oil Content and Oil Fatty Acid Composition to Evaluate Industrial Potential. Agrofor. Syst..

[47] Carvalho M.S., Corrêa P.C., Silva G.N., de Sousa A.H., Lopes L.M. (2023). Physico-Chemical Characteristics of the Oil from Macauba Kernels Stored after Drying at Different Temperatures. Rev. Cienc. Agron..

[48] Magalhães K.T., de Sousa Tavares T., Nunes C.A. (2020). The Chemical, Thermal and Textural Characterization of Fractions from Macauba Kernel Oil. Food Res. Int..

[49] de Souza E.F., Silva R.D.S., Santos M.N., de Andrade Silva C.A., Fiorucci A.R. (2020). Evaluation of Oxidative Stability, Fatty Acid Profile and Quality Physico-Chemical Parameters of Brazil Nut, Coconut and Palm Oils. Ciênc. Nat..

[50] Favaro S.P., Tapeti C.F., Miranda C.H.B., Ciaconini G., Amélia M.M.M., Roscoe R. (2017). Macauba (*Acrocomia aculeata*) Pulp Oil Quality Is Negatively Affected by Drying Fruits at 60 °C. Braz. Arch. Biol. Technol..

[51] Brand A.L.M., Silva A.C.R., Garrett R., Rezende C.M. (2024). Quantitative Lipidomics in Green Robusta Coffees from the Brazilian Amazon by LC-HRMS. Food Biosci..

[52] Silva A.C.R., da Silva C.C., Garrett R., Rezende C.M. (2020). Comprehensive Lipid Analysis of Green Arabica Coffee Beans by LC-HRMS/MS. Food Res. Int..

[53] Silva R.M.V., Brand L.M., Tinoco N.A.B., Freitas S.P., Rezende C.M. (2023). Bioactive Diterpenes and Serotonin Amides in Cold-Pressed Green Coffee Oil (*Coffea arabica* L.). J. Braz. Chem. Soc..

[54] Hartman L., Lago R.C. (1973). Rapid Preparation of Fatty Acid Methyl Esters from Lipids. Lab. Pract..

[55] Antoniassi R., Wilhelm A.E., Ferreira De Faria-Machado A., Maciel A.M., Humberto G., Bizzo R. (2018). Otimização do Método Hartman e Lago de Preparação de Ésteres Metílicos de Ácidos Graxos.

[56] Sun J., Hu P., Lyu C., Tian J., Meng X., Tan H., Dong W. (2022). Comprehensive Lipidomics Analysis of the Lipids in Hazelnut Oil during Storage. Food Chem..

[57] Tsugawa H., Cajka T., Kind T., Ma Y., Higgins B., Ikeda K., Kanazawa M., Vandergheynst J., Fiehn O., Arita M. (2015). MS-DIAL: Data-Independent MS/MS Deconvolution for Comprehensive Metabolome Analysis. Nat. Methods.

[58] Chong J., Wishart D.S., Xia J. (2019). Using MetaboAnalyst 4.0 for Comprehensive and Integrative Metabolomics Data Analysis. Curr. Protoc. Bioinform..

