# Supplementary material for: Quantitative Method for Analysis of Lipids by LC-HRMS and Fatty Acid Methyl Ester by GC-FID in Macauba (Acrocomia aculeata) Oils
Source: Plants (Basel). 2026 Jan 15;15(2):268. doi: 10.3390/plants15020268 (PMC12845416; doi:10.3390/plants15020268)
Supplement: Supplementary file 1 [file plants-15-00268-s001.zip › plants-4083332-supplementary.pdf]

Quantitative Method for Analysis of Lipids by LC-HRMS and Fatty Acid Methyl Ester by GC-FID in Macauba (*Acrocomia aculeata*) Oils

Eva Zopelario S. Ferro [1,2], Ana Laura M. Brand [1,3], Ricardo Sposina S. Teixeira [2] and Claudia M. Rezende [1,*]

[1] Aroma Analysis Laboratory, Institute of Chemistry, Federal University of Rio de Janeiro, Rio de Janeiro 21941-909, RJ, Brazil
[2] Bioethanol Laboratory, Institute of Chemistry, Federal University of Rio de Janeiro, Rio de Janeiro 21941-596, RJ, Brazil
[3] Metabolomics Laboratory (LabMeta-LADETEC), Institute of Chemistry, Federal University of Rio de Janeiro, Rio de Janeiro 21941-598, RJ, Brazil
* Correspondence: crezende@iq.ufrj.br

Supplementary Material

Section S1: Chromatogram of FAME profile of macauba *(Acrocomia aculeata)* pulp and seed oil control samples.

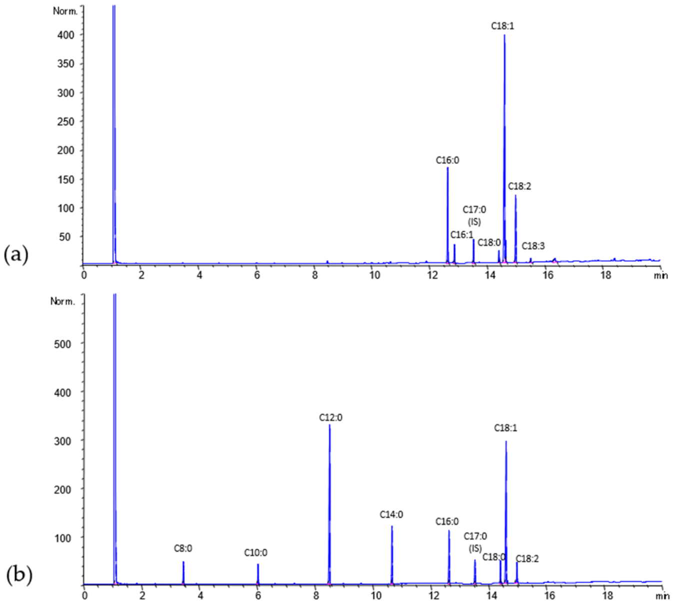

Figure S1: Chromatogram of the FAME profile obtained through GC-FID of macauba *(Acrocomia aculeata)* pulp (a) and seed (b) oil control sample.

Section S2: MS Dial parameters.

Table S1: Parameters used in the processing of data obtained by LC-HRMS in the MS-DIAL 4.7 software.

Parameters	Values

Data collection parameters	
MS1 tolerance	0.005 Da
MS1 tolerance	0.025 Da
Retention time range	0-25 min
Mass (*m*/*z*) range	140-2000 Da
Maximum charged number.	1
Peak detection parameters	
Minimun peak height	500,000
Smoothing method	LinearWeightedMovingAverage
Mass slice width.	0.1 Da
Smoothing level	3
Minimum peak width	5
Deconvolution parameters	
Sigma window value	0.4
MS2Dec amplitude cut off	10
Exclude after precursor ion	True
Keep isotope until	0.5
Keep the isotopic ions w/o MS2Dec.	True
MSP file and MS/MS identification setting	
Solvent type	Set true for $HCOONH_4$ for formate ammonium
Lipid classes	Set true for lipid classes: LPC; PI; PC; PE; DG; TG; PG; LPE; MG; SM., and ST
Retention time tolerance	100 min
Accurate mass tolerance (MS1)	0.005 Da
Accurate mass tolerance (MS2)	0.1 Da
Identification score cut off	70%
Adducts	
Positive ion mode	Set true for $[M + H]^+$, $[M + Na]^+$,

	[M + NH4]+, [M+H- H2O]+.
Negative ion mode	Set true for [M - H]-, [M + HCOO]- ,[M+ H - H2O]-
Alignment parameters setting	
Retention time tolerance	0.5 min
MS1 tolerance	0.015 Da
Retention time factor	0.5
MS1 factor	0.5
Peak count filter	0%
N% detected in at least one group	50%
Remove feature based on peak height fold-change	True
Sample average / blank average	5x
Gap filling by compulsion	False

Section S3: Trace Finder parameters.

Table S2: Parameters used in the processing of data obtained by LC-HRMS in the TraceFinder 4.0 software.

**Parameters**	**Values**
Processing parameters	
Qualitative peak processing template	Default
Backgroud substraction	Both sides of the peak
Number of scans to subtract	5
Stepoff value	5
Mass tolerance	5 ppm
Peak detection parameters	
Times	
Detection Type	Single detected

Window (sec)	30.00
View width (min)	1.00
Detect	
Sensitivity	Genesis
Detection method	Nearest RT
Smoothing	1
S/N threshold	2
Suitability	
Symmetry	Default
Peak classification	Default
Isotopes	
Isotopes	Not enabled

Section S4: Total ion chromatogram (TIC) of the lipid profile of quality control sample (QC POOL 3).

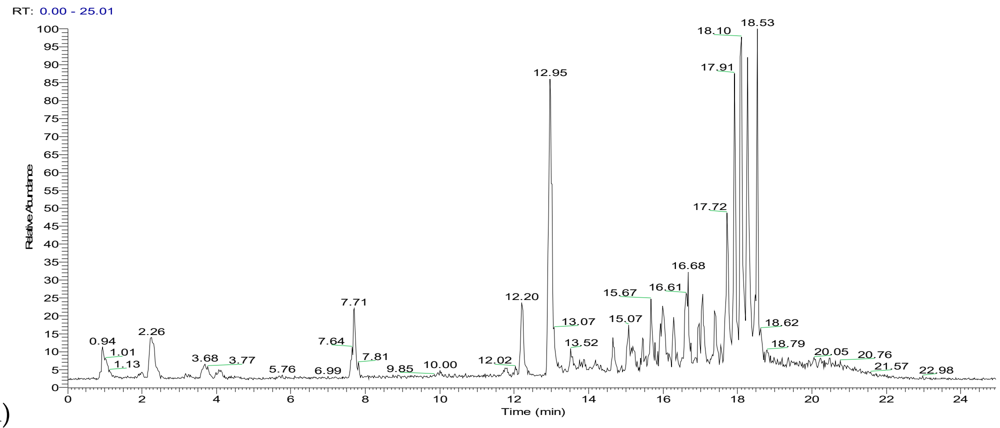

(a)

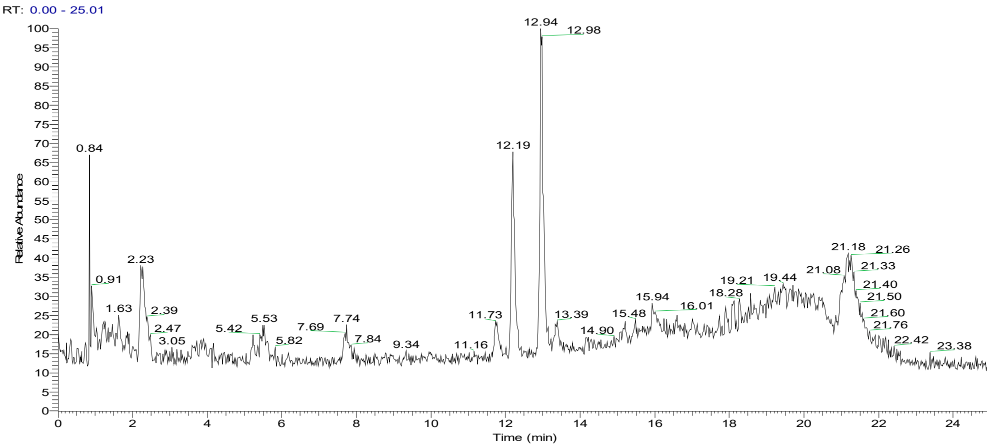

(b)

Figure S2: Total ion chromatogram (TIC) of the lipid profile of a quality control sample obtained through LC-HRMS on positive ion mode (a) and negative ion mode (b).

Section S5: Annotated compounds in the macauba *(Acrocomia aculeata)* oils commercial samples.

Table S3: Relative composition (%) of lipids species annotated in macauba *(Acrocomia aculeata)* commercial oils by LC-HRMS analysis.

Compound	RT	Adduct	*m/z* (Expected)	PCC1_1	PCC1_2	PC2	PC3	PC4	PC5	SCC1_1	SCC1_2	SC3	SC4	SC5
DG 26:0 | DG 12:0_14:0	11.78	$[M + NH4]^+$	502.44724	0	0	0	0	0	0	0.06	0.02	0	0	0
DG 32:0 | DG 16:0_16:0	15.89	$[M + NH4]^+$	586.5405	0.61	0.24	0.11	0.38	0.14	0.09	0.03	0.02	0.05	0.03	0.06
DG 34:0 | DG 16:0_18:0	16.57	$[M + NH4]^+$	614.5718	0.37	0.29	0.16	0.39	0.14	0.14	0.07	0.04	0.09	0.06	0.1
DG 34:1 | DG 16:0_18:1	15.93	$[M + NH4]^+$	612.55615	6.79	2.6	0.93	6.39	1.89	0.82	0.1	0.08	0.18	0.07	0.12
DG 34:2 | DG 16:0_18:2	15.43	$[M + NH4]^+$	610.5405	3.65	4.93	2.2	4.49	2.06	1.65	1.12	0.78	1.06	1.01	1.49
DG 36:0 | DG 18:0_18:0	17.02	$[M + NH4]^+$	642.6031	0.19	0.13	0.2	0.58	0.17	0.16	0.09	0.05	0.1	0.08	0.16
DG 36:1 | DG 18:0_18:1	16.57	$[M + NH4]^+$	640.58745	0.84	0.35	0.32	0.78	0.38	0.26	0.04	0.02	0.05	0.04	0.05
DG 36:2 | DG 18:1_18:1	15.97	$[M + NH4]^+$	638.5718	19.82	9.44	3.46	24.7	8.47	2.48	0.31	0.3	0.41	0.32	0.44
DG 36:3 | DG 18:1_18:2	15.2	$[M + NH4]^+$	636.55615	8.58	2.6	3.38	6.49	3.35	3.04	0.08	0.05	0.6	0.69	0.59
DG 36:4 | DG 18:2_18:2	14.19	$[M + NH4]^+$	634.5405	1	0.34	2.83	0.47	1.46	2.83	0.01	0.01	1.12	1.16	0.95
DG 36:5 | DG 18:2_18:3	13.19	$[M + NH4]^+$	632.52485	0.12	0.05	0.13	0.07	0.07	0.1	0	0	0.02	0.02	0.02

DG 40:2 | DG 22:0_18:2	17.09	[M + NH4]+	694.6344	0.02	0	0.06	0	0.03	0.05	0	0	0.01	0.02	0.01
TG 26:0 | TG 8:0_8:0_10:0	10.53	[M + NH4]+	516.42586	0.03	0.04	0.02	0.01	0.01	0.01	0.17	0.15	0.06	0.02	0.03
TG 28:0 | TG 8:0_10:0_10:0	12.28	[M + NH4]+	544.45717	0	0	0	0	0	0	0.98	0.79	0	0	0
TG 32:0 | TG 8:0_12:0_12:0	15.07	[M + NH4]+	600.51977	0.25	0.1	0.03	0.25	0.09	0.03	13.19	14.25	0.01	0	0
TG 34:0 | TG 10:0_12:0_12:0	16.02	[M + NH4]+	628.55107	0	0	0	0	0	0	13.15	14.22	0.03	0	0
TG 34:1 | TG 8:0_8:0_18:1	15.23	[M + NH4]+	626.53542	0.01	0.01	0.01	0.01	0.01	0.01	0.12	0.08	0	0	0
TG 36:0 | TG 12:0_12:0_12:0	16.62	[M + NH4]+	656.58237	0	0	0	0	0	0	13.91	15.83	0	0	0
TG 36:1 | TG 8:0_10:0_18:1	16.15	[M + NH4]+	654.56672	0.07	0.03	0.03	0.07	0.04	0.02	0.93	0.84	0.01	0.01	0.01
TG 36:2 | TG 8:0_10:0_18:2	15.43	[M + NH4]+	652.55107	0.01	0	0.01	0	0.01	0	0.02	0.03	0	0	0
TG 38:0 | TG 12:0_12:0_14:0	17.06	[M + NH4]+	684.61367	0	0	0	0	0	0	9.58	8.54	0	0	0
TG 38:1 | TG 8:0_12:0_18:1	16.67	[M + NH4]+	682.59802	0.14	0.07	0.08	0.22	0.18	0.09	3.93	3	0.02	0.02	0.02
TG 38:2 | TG 8:0_12:0_18:2	16.18	[M + NH4]+	680.58237	0.07	0.03	0.02	0.07	0.04	0.01	0.18	0.22	0	0	0
TG 40:0 | TG 12:0_12:0_16:0	17.4	[M + NH4]+	712.64497	0	0	0.04	0.29	0.15	0.04	4.41	4.13	0	0	0
TG 40:1 | TG 10:0_12:0_18:1	17.09	[M + NH4]+	710.62932	0.23	0.13	0.1	0.34	0.16	0.12	2.82	2.4	0.03	0.03	0.03
TG 40:2 | TG 8:0_14:0_18:2	16;75	[M + NH4]+	708.61367	0.07	0.04	0.02	0.04	0.03	0.02	0.17	0.2	0	0	0
TG 42:0 | TG 12:0_14:0_16:0	17.67	[M + NH4]+	740.67627	0.18	0.16	0.07	0.19	0.16	0.09	2.35	1.8	0.08	0.06	0.08
TG 42:1 | TG 12:0_12:0_18:1	17.4	[M + NH4]+	738.66062	0.38	0.28	0.2	0.75	0.41	0.25	5.52	5.76	0.09	0.08	0.08
TG 42:2 | TG 8:0_16:0_18:2	17.12	[M + NH4]+	736.64497	0.15	0.13	0.09	0.19	0.13	0.1	0.7	0.84	0.04	0.03	0.03
TG 44:0 | TG 12:0_14:0_18:0	17.89	[M + NH4]+	768.70757	0.39	0.35	0.13	0.32	0.14	0.1	0.87	0.68	0.14	0.07	0.14
TG 44:2 | TG 8:0_18:1_18:1	17.43	[M + NH4]+	764.67627	0.22	0.24	0.16	0.38	0.24	0.16	2.04	1.9	0.05	0.08	0.04
TG 44:3 | TG 8:0_18:1_18:2	17.14	[M + NH4]+	762.66062	0.03	0.03	0.06	0.05	0.06	0.04	0.32	0.33	0.03	0.03	0.03

TG 44:4 ǀ TG 8:0_18:2_18:2	16.82	[M + NH4]+	760.64497	0	0.01	0.02	0	0.02	0.03	0.05	0.06	0.03	0.04	0.03
TG 45:3;O ǀ TG 18:1_18:1_9:1;O	16.74	[M + NH4]+	792.67118	0.03	0	0	0	0	0	0	0	0	0	0
TG 46:0 ǀ TG 14:0_16:0_16:0	18.1	[M + NH4]+	796.73887	0.5	0.56	0.21	0.41	0.17	0.12	0.51	0.34	0.16	0.1	0.2
TG 46:1 ǀ TG 12:0_16:0_18:1	17.88	[M + NH4]+	794.72322	0.67	0.8	0.25	0.86	0.51	0.31	2.52	2.63	0.21	0.12	0.22
TG 46:2 ǀ TG 14:0_16:1_16:1	17.67	[M + NH4]+	792.70757	0.1	0.08	0.03	0.16	0.17	0.04	1.22	1.05	0	0	0
TG 48:0 ǀ TG 16:0_16:0_16:0	18.3	[M + NH4]+	824.77017	0.85	0.56	0.25	0.58	0.29	0.19	0.34	0.25	0.17	0.19	0.21
TG 48:1 ǀ TG 14:0_16:0_18:1	18.11	[M + NH4]+	822.75452	1.06	1.18	0.42	0.75	0.53	0.19	1.66	1.29	0.43	0.32	0.2
TG 48:2 ǀ TG 12:0_18:1_18:1	17.88	[M + NH4]+	820.73887	0.42	0.46	0.12	1.49	0.59	0.27	3.9	4.62	0	0	0
TG 48:3 ǀ TG 12:0_18:1_18:2	17.7	[M + NH4]+	818.72322	0	0	0.08	0.19	0.2	0.16	0.3	0.93	0	0	0
TG 50:0 ǀ TG 16:0_16:0_18:0	18.46	[M + NH4]+	852.80147	0.38	0.35	0.14	0.27	0.17	0.11	0.06	0.06	0.06	0.03	0.08
TG 50:1 ǀ TG 16:0_16:0_18:1	18.29	[M + NH4]+	850.78582	4.23	3.54	0.84	2.2	1.32	0.79	1.01	0.86	0.68	0.63	0.7
TG 50:2 ǀ TG 16:0_16:0_18:2	18.14	[M + NH4]+	848.77017	0	0	1.32	1.64	1.48	1.22	1.48	1.32	0	1.27	1.27
TG 52:1 ǀ TG 16:0_18:0_18:1	18.46	[M + NH4]+	878.81712	1.29	1.63	0.71	0.78	0.72	0.66	0.42	0.3	0.66	0.62	0.69
TG 52:2 ǀ TG 16:0_18:1_18:1	18.28	[M + NH4]+	876.80147	13.6	16.75	4.54	10.23	6.37	4.84	2.64	2.54	4.08	4.24	4.29
TG 52:4;O ǀ TG 16:0_18:2_18:2;O	17.29	[M + NH4]+	888.76508	0.07	0.05	0.04	0.01	0.04	0.05	0	0	0.17	0.17	0.15
TG 52:4 ǀ TG 16:0_18:2_18:2	17.95	[M + NH4]+	872.77017	3	4.61	6.9	1.33	5.72	7.07	0.27	0.25	9.75	9.31	8.82
TG 52:5 ǀ TG 16:0_18:2_18:3	17.77	[M + NH4]+	870.75452	0.56	0.87	0.58	0.17	0.55	0.59	0	0	0.52	0.54	0.57
TG 54:0 ǀ TG 18:0_18:0_18:0	18.81	[M + NH4]+	908.86407	0.25	0.32	0.15	0.25	0.17	0.15	0.09	0.07	0.16	0.13	0.15
TG 54:1 ǀ TG 18:0_18:0_18:1	18.63	[M + NH4]+	906.84842	0.35	0.44	0.43	0.32	0.4	0.5	0.22	0.16	0.49	0.42	0.42
TG 54:2 ǀ TG 18:0_18:1_18:1	18.45	[M + NH4]+	904.83277	2.35	4.28	3.4	1.87	3	3.61	1.02	0.8	3.34	3.11	3.1
TG 54:3 ǀ TG 18:1_18:1_18:1	18.27	[M + NH4]+	902.81712	13.79	22.68	11.7	18.87	13.73	12.99	3.11	3.35	9.55	9.81	9.77

TG 54:4;O I TG 18:1_18:2_18:1;O	17.48	[M + NH4]+	916.79638	0.51	0.23	0.08	0.18	0.14	0.12	0.01	0	0.1	0.11	0.11
TG 54:4 I TG 18:1_18:1_18:2	18.11	[M + NH4]+	900.80147	7.42	10.95	14.9	6.54	11.89	14.76	0.9	0.92	16.67	15.88	15.66
TG 54:5 I TG 18:1_18:2_18:2	17.94	[M + NH4]+	898.78582	2.79	4.48	16.48	1.75	13.49	15.91	0.43	0.38	19.69	18.91	18.2
TG 54:6 I TG 18:2_18:2_18:2	17.73	[M + NH4]+	896.77017	0.71	1.17	16.82	0.4	14.59	16.81	0.14	0.14	22.12	23.49	24.42
TG 54:7 I TG 18:2_18:2_18:3	17.55	[M + NH4]+	894.75452	0.01	0.17	0.99	0.04	0.77	1.26	0	0	1.42	1.57	1.38
TG 56:1 I TG 16:0_22:0_18:1	18.8	[M + NH4]+	934.87972	0.16	0.22	0.19	0.13	0.16	0.22	0.08	0.05	0.22	0.2	0.2
TG 56:2 I TG 20:0_18:1_18:1	18.63	[M + NH4]+	932.86407	0.23	0.32	0.46	0.23	0.4	0.55	0.12	0.1	0.65	0.6	0.55
TG 56:3 I TG 18:1_18:1_20:1	18.45	[M + NH4]+	930.84842	0.19	0.36	0.48	0.22	0.43	0.52	0.08	0.07	0.51	0.46	0.48
TG 58:2 I TG 22:0_18:1_18:1	18.79	[M + NH4]+	960.89537	0.12	0.16	0.65	0.09	0.51	0.87	0.05	0.03	0.68	0.7	0.6
TG 58:3 I TG 22:0_18:1_18:2	18.64	[M + NH4]+	958.87972	0.05	0.06	0.57	0.04	0.41	0.69	0.01	0.01	0.9	0.83	0.79
TG 58:4 I TG 22:0_18:2_18:2	18.48	[M + NH4]+	956.86407	0	0	0.71	0	0.53	0.82	0	0	1.35	1.34	1.41
TG 60:2 I TG 24:0_18:1_18:1	18.96	[M + NH4]+	988.92667	0.07	0.09	0.21	0.06	0.17	0.23	0.03	0.02	0.18	0.2	0.18
TG 60:3 I TG 24:0_18:1_18:2	18.8	[M + NH4]+	986.91102	0.03	0.05	0.2	0.02	0.15	0.27	0.01	0.01	0.25	0.27	0.23
TG 60:4 I TG 24:0_18:2_18:2	18.65	[M + NH4]+	984.89537	0.01	0.01	0.26	0.01	0.17	0.31	0	0	0.48	0.47	0.41

Table S4: Concentration in g 100g^{-1} macauba *(Acrocomia aculeata)* oils of annotated lipid species obtained by LC-HRMS analysis.

Compound	RT	Adduct	*m/z* (Expected)	PCC1_1	PCC1_2	PC2	PC3	PC4	PC5	SCC1_1	SCC1_2	SC3	SC4	SC5
DG 26:0 I DG 12:0_14:0	11.78	[M + NH4]+	502.44724	-	-	-	-	-	-	n.q.	n.q.	-	-	-
DG 32:0 I DG 16:0_16:0	15.89	[M + NH4]+	586.5405	0.337	n.q.	n.q.	0.244	n.q.	n.q.	n.q.	n.q.	n.q.	n.q.	n.q.
DG 34:0 I DG 16:0_18:0	16.57	[M + NH4]+	614.5718	0.202	n.q.	n.q.	0.248	n.q.	0.246	n.q.	n.q.	n.q.	n.q.	n.q.
DG 34:1 I DG 16:0_18:1	15.93	[M + NH4]+	612.55615	3.737	1.367	1.202	4.1	1.996	1.415	0.230	0.252	0.364	n.q.	0.212

DG 34:2 ǀ DG 16:0_18:2	15.43	[M + NH4]+	610.5405	2.009	2.585	2.823	2.882	2.170	2.849	2.609	2.418	2.083	2.656	2.537
DG 36:0 ǀ DG 18:0_18:0	17.02	[M + NH4]+	642.6031	n.q.	n.q.	0.257	0.375	n.q.	0.275	n.q.	n.q.	n.q.	n.q.	0.275
DG 36:1 ǀ DG 18:0_18:1	16.57	[M + NH4]+	640.58745	0.464	n.q.	0.411	0.503	0.399	0.442	n.q.	n.q.	n.q.	n.q.	n.q.
DG 36:2 ǀ DG 18:1_18:1	15.97	[M + NH4]+	638.5718	10.908	4.956	4.447	15.845	8.933	4.269	0.714	0.926	0.812	0.842	0.753
DG 36:3 ǀ DG 18:1_18:2	15.2	[M + NH4]+	636.55615	4.723	1.366	4.345	4.164	3.538	5.239	n.q.	n.q.	1.190	1.819	1.002
DG 36:4 ǀ DG 18:2_18:2	14.19	[M + NH4]+	634.5405	0.550	n.q.	3.640	0.299	1.538	4.868	n.q.	n.q.	2.210	3.048	1.628
TG 28:0 ǀ TG 8:0_10:0_10:0	12.28	[M + NH4]+	544.45717	-	-	-	-	-	-	2.286	2.466	-	-	-
TG 32:0 ǀ TG 8:0_12:0_12:0	15.07	[M + NH4]+	600.51977	n.q.	n.q.	n.q.	n.q.	n.q.	n.q.	30.704	44.291	n.q.	-	-
TG 34:0 ǀ TG 10:0_12:0_12:0	16.02	[M + NH4]+	628.55107	-	-	-	-	-	-	30.6065	44.180	n.q.	-	-
TG 36:0 ǀ TG 12:0_12:0_12:0	16.62	[M + NH4]+	656.58237	-	-	-	-	-	-	32.396	49.203	-	-	-
TG 36:1 ǀ TG 8:0_10:0_18:1	16.15	[M + NH4]+	654.56672	n.q.	n.q.	n.q.	n.q.	n.q.	n.q.	2.1705	2.622	n.q.	n.q.	n.q.
TG 38:0 ǀ TG 12:0_12:0_14:0	17.06	[M + NH4]+	684.61367	-	-	-	-	-	-	22.307	26.551	-	-	-
TG 38:1 ǀ TG 8:0_12:0_18:1	16.67	[M + NH4]+	682.59802	n.q.	n.q.	n.q.	n.q.	n.q.	n.q.	9.145	9.329	n.q.	n.q.	n.q.
TG 40:0 ǀ TG 12:0_12:0_16:0	17.4	[M + NH4]+	712.64497	-	-	n.q.	n.q.	n.q.	n.q.	10.268	12.832	-	-	-
TG 40:1 ǀ TG 10:0_12:0_18:1	17.09	[M + NH4]+	710.62932	n.q.	n.q.	n.q.	n.q.	n.q.	n.q.	6.575	7.455	n.q.	n.q.	n.q.
TG 42:0 ǀ TG 12:0_14:0_16:0	17.67	[M + NH4]+	740.67627	n.q.	n.q.	n.q.	n.q.	n.q.	n.q.	5.477	5.588	n.q.	n.q.	n.q.
TG 42:1 ǀ TG 12:0_12:0_18:1	17.4	[M + NH4]+	738.66062	n.q.	n.q.	n.q.	n.q.	n.q.	n.q.	12.859	17.910	n.q.	n.q.	n.q.
TG 42:2 ǀ TG 8:0_16:0_18:2	17.12	[M + NH4]+	736.64497	n.q.	n.q.	n.q.	n.q.	n.q.	n.q.	1.630	2.624	n.q.	n.q.	n.q.
TG 44:0 ǀ TG 12:0_14:0_18:0	17.89	[M + NH4]+	768.70757	n.q.	n.q.	n.q.	n.q.	n.q.	n.q.	2.027	2.107	n.q.	n.q.	n.q.
TG 44:2 ǀ TG 8:0_18:1_18:1	17.43	[M + NH4]+	764.67627	n.q.	n.q.	n.q.	n.q.	n.q.	n.q.	4.755	5.909	n.q.	n.q.	n.q.
TG 44:3 ǀ TG 8:0_18:1_18:2	17.14	[M + NH4]+	762.66062	n.q.	n.q.	n.q.	n.q.	n.q.	n.q.	n.q.	1.039	n.q.	n.q.	n.q.

TG 46:0ǀTG 14:0_16:0_16:0	18.1	[M + NH4]+	796.73887	n.q.	n.q.	n.q.	n.q.	n.q.	n.q.	1.181	1.054	n.q.	n.q.	n.q.
TG 46:1ǀTG 12:0_16:0_18:1	17.88	[M + NH4]+	794.72322	n.q.	n.q.	n.q.	n.q.	n.q.	n.q.	5.871	8.179	n.q.	n.q.	n.q.
TG 46:2ǀTG 14:0_16:1_16:1	17.67	[M + NH4]+	792.70757	n.q.	n.q.	n.q.	n.q.	n.q.	n.q.	2.843	3.266	-	-	-
TG 48:1ǀTG 14:0_16:0_18:1	18.11	[M + NH4]+	822.75452	n.q.	n.q.	n.q.	n.q.	n.q.	n.q.	3.873	4.008	n.q.	n.q.	n.q.
TG 48:2ǀTG 12:0_18:1_18:1	17.88	[M + NH4]+	820.73887	n.q.	n.q.	n.q.	n.q.	n.q.	n.q.	9.086	14.365	-	-	-
TG 48:3ǀTG 12:0_18:1_18:2	17.7	[M + NH4]+	818.72322	-	-	n.q.	n.q.	n.q.	n.q.	n.q.	2.886	-	-	-
TG 50:1ǀTG 16:0_16:0_18:1	18.29	[M + NH4]+	850.78582	2.329	1.859	1.075	1.412	1.393	1.364	2.347	2.663	1.348	1.657	1.197
TG 50:2ǀTG 16:0_16:0_18:2	18.14	[M + NH4]+	848.77017	-	-	1.701	1.049	1.558	2.107	3.453	4.089	-	3.347	2.162
TG 52:1ǀTG 16:0_18:0_18:1	18.46	[M + NH4]+	878.81712	n.q.	n.q.	n.q.	n.q.	n.q.	1.144	0.977	n.q.	1.293	1.632	1.175
TG 52:2ǀTG 16:0_18:1_18:1	18.28	[M + NH4]+	876.80147	7.484	8.790	5.831	6.567	6.715	8.338	6.158	7.879	8.040	11.138	7.322
TG 52:4ǀTG 16:0_18:2_18:2	17.95	[M + NH4]+	872.77017	1.651	2.419	8.866	n.q.	6.0282	12.168	n.q.	n.q.	19.235	24.454	15.048
TG 52:5ǀTG 16:0_18:2_18:3	17.77	[M + NH4]+	870.75452	n.q.	n.q.	n.q.	n.q.	n.q.	n.q.	-	-	1.032	1.409	0.975
TG 54:1ǀTG 18:0_18:0_18:1	18.63	[M + NH4]+	906.84842	n.q.	n.q.	n.q.	n.q.	n.q.	n.q.	n.q.	n.q.	0.97	1.1	n.q.
TG 54:2ǀTG 18:0_18:1_18:1	18.45	[M + NH4]+	904.83277	1.292	2.249	4.364	1.200	3.159	6.225	2.382	2.497	6.584	8.182	5.292
TG 54:3ǀTG 18:1_18:1_18:1	18.27	[M + NH4]+	902.81712	7.588	11.905	15.045	12.107	14.480	22.371	7.242	10.414	18.840	25.779	16.672
TG 54:4ǀTG 18:1_18:1_18:2	18.11	[M + NH4]+	900.80147	4.084	5.747	19.156	4.195	12.539	25.4232	2.097	2.861	32.881	41.735	26.73
TG 54:5ǀTG 18:1_18:2_18:2	17.94	[M + NH4]+	898.78582	1.534	2.352	21.191	1.120	14.222	27.405	1.003	1.168	38.829	49.687	31.054
TG 54:6ǀTG 18:2_18:2_18:2	17.73	[M + NH4]+	896.77017	n.q.	n.q.	21.621	n.q.	15.385	28.953	n.q.	n.q.	43.632	61.738	41.68
TG 54:7ǀTG 18:2_18:2_18:3	17.55	[M + NH4]+	894.75452	n.q.	n.q.	1.275	n.q.	n.q.	2.163	n.q.	n.q.	2.802	4.115	2.363
TG 56:2ǀTG 20:0_18:1_18:1	18.63	[M + NH4]+	932.86407	n.q.	n.q.	n.q.	n.q.	n.q.	n.q.	n.q.	n.q.	1.291	1.588	0.940
TG 56:3ǀTG 18:1_18:1_20:1	18.45	[M + NH4]+	930.84842	n.q.	n.q.	n.q.	n.q.	n.q.	n.q.	n.q.	n.q.	0.999	1.203	n.q.

TG 58:2 ǀ TG 22:0_18:1_18:1	18.79	$[M + NH4]^+$	960.89537	n.q.	n.q.	n.q.	n.q.	n.q.	1.496	n.q.	n.q.	1.336	1.832	1.029
TG 58:3 ǀ TG 22:0_18:1_18:2	18.64	$[M + NH4]^+$	958.87972	n.q.	n.q.	n.q.	n.q.	n.q.	1.193	n.q.	n.q.	1.782	2.192	1.340
TG 58:4 ǀ TG 22:0_18:2_18:2	18.48	$[M + NH4]^+$	956.86407	-	-	n.q.	-	n.q.	1.405	-	-	2.665	3.508	2.398
TG 60:4 ǀ TG 24:0_18:2_18:2	18.65	$[M + NH4]^+$	984.89537	n.q.	n.q.	n.q.	n.q.	n.q.	n.q.	n.q.	n.q.	0.946	1.237	n.q.

Dash (-): not detected; n.q.: .: detected but not quantified. Compounds not listed in this table are below LOQ.
